# Atomic Scale Engineering of Multivalence‐State Palladium Photocatalyst for Transfer Hydrogenation with Water as a Proton Source

**DOI:** 10.1002/adma.202504108

**Published:** 2025-05-22

**Authors:** En Zhao, Wenjing Kong, Giorgio Zoppellaro, Yue Yang, Bing Nan, Lina Li, Wengjun Zhang, Zhaohui Chen, Aristides Bakandritsos, Zhu‐Jun Wang, Matthias Beller, Radek Zbořil, Zupeng Chen

**Affiliations:** ^1^ National Key Laboratory for the Development and Utilization of Forest Food Resources, Jiangsu Co‐Innovation Center of Efficient Processing and Utilization of Forest Resources, International Innovation Center for Forest Chemicals and Materials Nanjing Forestry University Longpan Road 159 Nanjing 210037 China; ^2^ Regional Centre of Advanced Technologies and Materials, Czech Advanced Technology and Research Institute (CATRIN) Palacký University Olomouc Šlechtitelů 27 Olomouc 783 71 Czech Republic; ^3^ Nanotechnology Centre Centre for Energy and Environmental Technologies VSB‐Technical University of Ostrava 17. listopadu 2172/15 Ostrava‐Poruba 708 00 Czech Republic; ^4^ School of Physical Science and Technology Shanghai Tech University Shanghai 201210 P. R. China; ^5^ Shanghai Synchrotron Radiation Facility Shanghai Advanced Research Institute Zhangheng Road 293 Shanghai 201204 P. R. China; ^6^ Leibniz‐Institute for Catalysis Albert‐Einstein‐Straβe 29a 18059 Rostock Germany

**Keywords:** carbon nitride, hydride transfer, palladium, photocatalysis, selective hydrogenation

## Abstract

Hydrogenation reactions are fundamental in the fine chemical, pharmaceutical, and petrochemical industries, however heavily relying on H_2_ gas at high temperatures and pressures, incurring large energy and carbon costs. Photocatalytic transfer hydrogenation, using water as a proton source, offers a greener alternative, but existing photocatalysts often suffer from modest yields, limited selectivity, and narrow substrate scope. Additionally, they often require co‐activation, such as Mg‐activated water or non‐sustainable hydrogen feeds. Here, a photocatalyst is introduced that offers high yields and selectivities across a broad spectrum of organic compounds. The developed photocatalyst is a multivalence palladium superstructure with ultrasmall Pd^0^ nanoparticles enveloped by isolated Pd^2+^/Pd^4+^ atoms within a carbon‐nitride matrix. Mechanistic studies reveal that the redox‐flexible Pd single atoms, with triethylamine as an electronic modulator, attract photogenerated holes for water oxidation, while Pd^0^ nanoparticles facilitate hydrogen transfer to the unsaturated bonds of the organic molecules. The cooperative and dynamic behavior of Pd centers during catalysis, involving transitions among Pd^+2^, Pd^+3^, and Pd^+4^ states, is validated using operando electron paramagnetic resonance spectroscopy. This multivalent palladium catalyst represents a conceptual advance in photocatalytic transfer hydrogenation with water as a hydrogen source, holding promise for sustainable hydrogenation processes in the chemical industry.

## Graphical abstract

Photocatalytic transfer hydrogenation is achieved over a multivalence‐state palladium photocatalyst, in which protons generated from photocatalytic water splitting are added to unsaturated double bonds. The *operando* light‐induced Electron Paramagnetic Resonance (EPR) spectroscopy confirms the synergistic action between Pd single atoms and nanoparticles, in which the H^‐^ produced by palladium nanoparticles migrate to single atoms where the substrate is located, resulting in hydrogenation reaction.

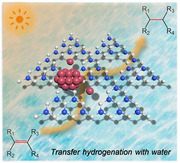

## 1 Introduction

**Scheme 1:**
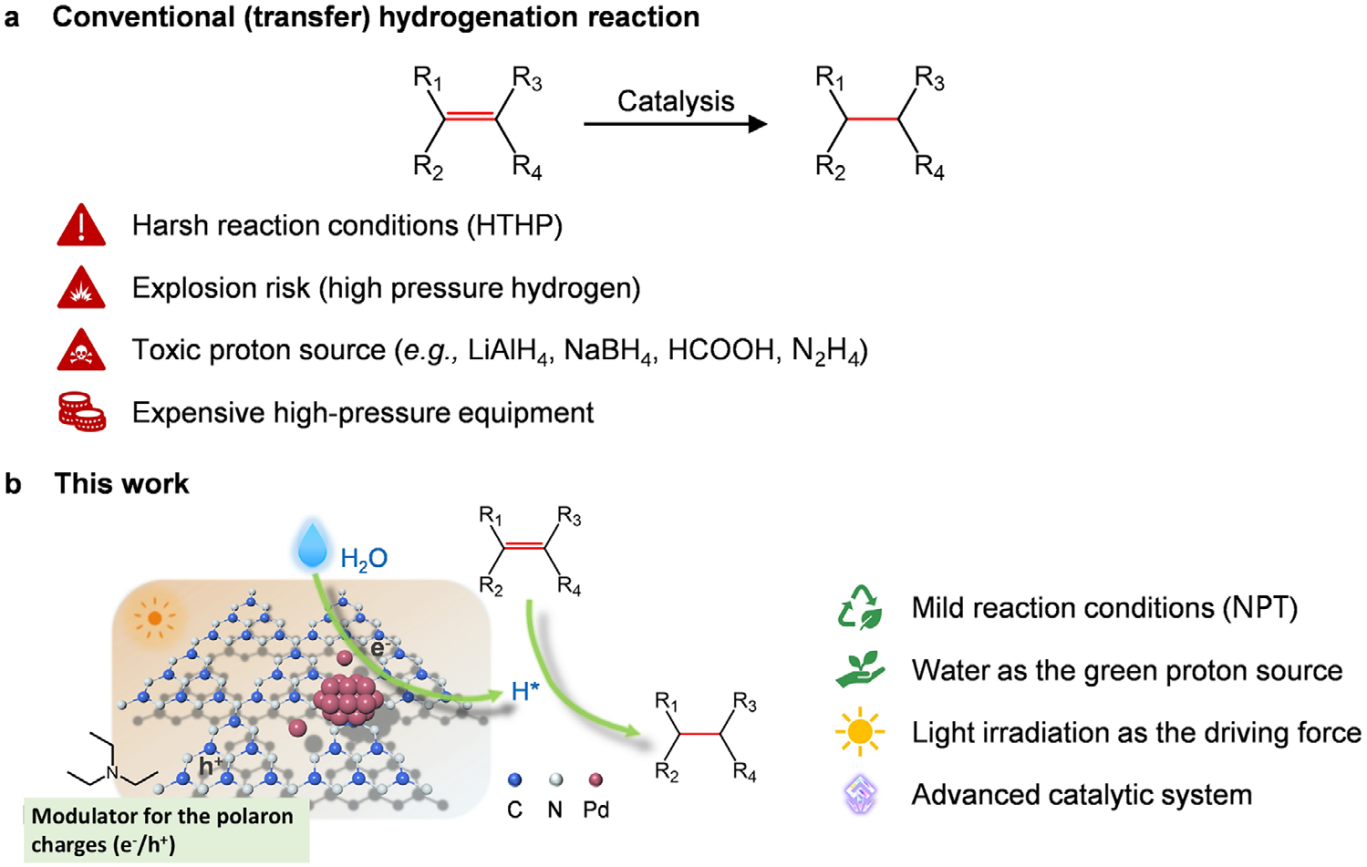
a) Conventional (transfer) hydrogenation process. b) Photocatalytic selective transfer hydrogenation using H_2_O as a proton source in this work. HTHP = High temperature and high pressure; NPT = Normal pressure and temperature. Atom color codes: blue, carbon; white, nitride; red, palladium.

Hydrogenation reactions are chemical processes of great importance for the chemical industry because they form the basis for synthesizing several commodity chemicals, fine chemicals, and pharmaceuticals.^[^ ^1^, ^2^, ^3^ ^]^ Two different approaches have been extensively implemented for such purpose,^[^ ^4^, ^5^ ^]^ One approach relies on the direct hydrogenation (DH) of substrates and the other employs transfer hydrogenation (TH) (**Scheme** **1**a). Direct hydrogenation involves the presence of high operational temperatures and highly pressurized H_2_ gas in the reactor chamber,^[^ ^6^, ^7^ ^]^ while transfer hydrogenation reactions require milder conditions and the presence of molecular species other than molecular hydrogen as the H source.^[^ ^8^ ^]^ Irrespective of the chemical route selected for the hydrogenation process, the development of effective and highly tailored catalytic systems is crucial.^[^ ^9^, ^10^ ^]^

For this reason, the last decade has witnessed an increased research interest in the discovery of more efficient, greener, and thus sustainable catalysts that can convert key raw and waste materials into high‐value chemicals.^[^ ^11^, ^12^, ^13^, ^14^, ^15^, ^16^, ^17^ ^]^ The target is to gradually free the chemical industry from its current dependence on non‐renewable energy sources. In such a scenario, the conventional thermal hydrogenation technology seems to have reached functional limits,^[^ ^5^ ^]^ as it is an energy‐hungry process that uses harsh operational conditions (high‐pressure, high‐temperature) and flammable H_2_. These factors pose technological challenges to the chemical plants that must meet strict national regulations to ensure safety, low environmental impacts, and long‐term sustainability. Transfer hydrogenation reactions benefit from the use of milder operational conditions compared to direct hydrogenation and thus it is clearly the safer choice to implement in the planned conversion/renovation of chemical infrastructures,^[^ ^18^, ^19^, ^20^, ^21^, ^22^, ^23^ ^]^ Photocatalytic transfer hydrogenation using water or alcohols as the hydrogen source under mild reaction conditions is undoubtedly the greenest pathway to directly hydrogenate various unsaturated bonds.^[^ ^24^ ^]^ Among the variety of industrially valuable substrates, the selective hydrogenation of *α*,*β*‐unsaturated carbonyl compounds to the corresponding carbonyl compounds, which are key molecules/intermediates for the fine chemicals and pharma industry, remains particularly difficult to achieve, irrespective of the pathway chosen (DH or TH process).^[^ ^5^, ^7^, ^8^, ^25^, ^26^ ^]^

The difficulty arises from low yields and selectivity (e.g., the competition in the reduction of both C═C and C═O bonds).^[^ ^1^, ^2^ ^]^ The other issue is the high energy barrier for water splitting, resulting in the use of less sustainable than water proton sources (e.g., alcohols). Thus, Guo et al. reported the successful photocatalytic transfer hydrogenation of *α*,*β*‐unsaturated aldehydes to the corresponding unsaturated alcohols by using 2‐propanol as the hydrogen source and Au/SiC nanocomposite as photocatalyst at room temperature.^[^ ^27^ ^]^ Other alternatives cover the use of co‐activating systems to improve the photocatalytic transfer hydrogenation process. Thus, photocatalytic transfer hydrogenation reaction of *α*,*β*‐unsaturated aldehydes to the corresponding aldehydes was achieved using Au─Pd/ultrathin SnNb_2_O_6_ nanosheets, but with an external feed of flammable H_2_.^[^ ^28^ ^]^ Sasson et al. reported the photoreduction of alkenes and nitro compounds via Mg‐activated water as the hydrogen source and a palladium‐based carbon nitride (Pd‐g‐C_3_N_4_) as a catalyst; however, the presence of magnesium powder in the reaction mixture containing the Pd catalyst posed operational limitations, due to the associated safety risks arising from dust explosion.^[^ ^29^ ^]^ Our group has earlier discovered a carbon‐nitride‐supported palladium single‐atom heterogeneous catalyst (Pd SAC) with higher performance in photocatalytic water‐donating transfer hydrogenation compared to its nanoparticle counterparts (Pd NP).^[^ ^24^ ^]^ Despite this significant progress, the efficiency of Pd SAC, in terms of conversion of unsaturated C═C bonds and ability to apply it for a broad portfolio of substrates, still remains limited. The development of efficient, selective, and universal photocatalysts with novel well‐defined modes of action, and enabling to convert of unsaturated C═C bonds to saturated ones for a broad scale of organic compounds, is still one of the biggest challenges. Addressing this challenge would move the photocatalytic transfer hydrogenation process with water as a proton source into the real industrial practice, offering the potential to substitute energy‐demanding thermal catalysis with molecular hydrogen thus having an immediate technological impact.

In this work, we show the unique design and performance of an advanced catalytic system (labeled as Pd‐mpg‐CN) comprised of multivalence‐state Pd(0/2+/4+) centers firmly entrapped into photocatalytic carbon nitride support (Scheme 1b). The resulting Pd‐mpg‐CN system acted as a universal catalyst enabling highly selective activation and photocatalytic transfer hydrogenation of the desired functional group (C═C) in the broad scale of organic substrates toward corresponding ketones, aldehydes, esters, amides, carboxylic acids, nitriles, aromatic olefins, ethers, or alcohols; moreover, with impressive yields and selectivities. For example, an industrially important process of chalcone hydrogenation to 1,3‐diphenylpropan‐1‐one, was obtained in 95% conversion and >99% selectivity in ≈4 h using water and Pd‐mpg‐CN photocatalyst. With the use of a broad battery of advanced synchrotron, microscopic, and spectroscopic tools (e.g., extended X‐ray absorption fine structure (EXAFS), X‐ray absorption near edge structure (XANES), high‐angle annular dark field scanning TEM (HAADF‐STEM), and *operando* electron paramagnetic resonance spectroscopy (EPR), we clearly proved a well‐defined mechanism based on the synergistic action of various catalytic centers embedded in the carbon nitride matrix. The effectiveness and the tailored selectivity result from combination of the following factors: i) cooperativity between photogenerated electrons and holes, from the carbon nitride support photoexcitation, that are effectively delivered to ii) Pd single atoms, site‐specific for substrates binding, and iii) Pd nanoparticles, where hydride anions are in situ generated from H^+^. The observed synergy in electron‐hole excitation, substrate and water activation, and delivery to the substrate of reducing equivalents, as experimentally probed during turn‐over, is unprecedented, and closely mimics the behavior seen in natural counterparts, for the hydrogenation reactions catalyzed by oxidoreductases with cofactors, e.g., nicotinamide adenine dinucleotide, nicotinamide adenine dinucleotide phosphate. This unprecedented mechanism, the universal efficiency of photocatalyst for a broad scale of substrates including industrially important compounds possess a significant step toward the real implementation of photocatalytic transfer hydrogenation with water as a proton source into technological practice.

## 2 Results and Discussion

### 2.1 Physicochemical Properties

**Figure 1:**
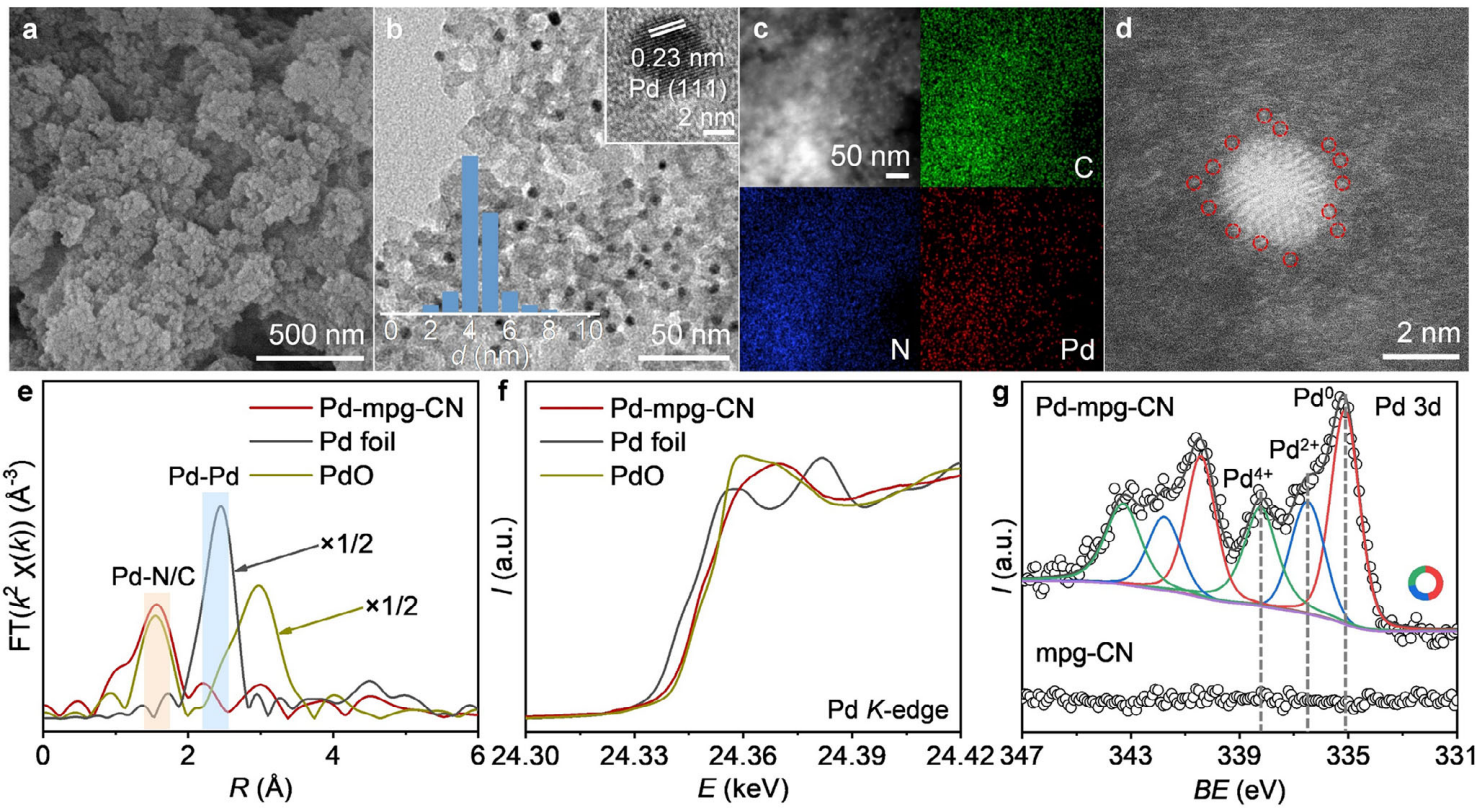
a) SEM, b) HRTEM, c) HAADF‐STEM with the corresponding elemental maps (i.e., C, N, and Pd), and d) AC‐HAADF‐STEM images of Pd‐mpg‐CN. Isolated Pd single atoms are highlighted by red circles. e) Fourier transform of *k* ^2^‐weighted Pd *K*‐edge EXAFS spectra and f) Pd *K*‐edge XANES spectra Pd‐mpg‐CN. Pd foil and PdO were applied for comparison. g) Pd 3*d* XPS spectra of mpg‐CN and Pd‐mpg‐CN. The donut plot in (g) indicates the relative distribution of Pd^0^ (red), Pd^2+^ (blue), and Pd^4+^ (green).

The mpg‐CN was synthesized by the polymerization of cyanamide exploiting colloidal silica as a template and the Pd species were deposited uniformly by the adsorption‐reduction method (Pd‐mpg‐CN) with a metal loading of 2.9 wt.% as determined by the inductively coupled plasma optical emission spectrometry (ICP‐OES). The intrinsic crystal patterns of mpg‐CN and Pd‐mpg‐CN were confirmed by X‐ray diffraction (XRD) analysis (Figure S1a, Supporting Information). Both of these catalysts exhibited two peaks at 13.2 and 27.3°, corresponding to the in‐plane repeating motifs and interlayer stacking of the graphitic carbon nitride carrier, respectively.^[^ ^29^, ^30^, ^31^ ^]^ Pd‐mpg‐CN additionally exhibited the diffraction peaks of Pd at 39.3 and 45.5°, corresponding to the characteristic (111) and (200) crystal planes of Pd(0) NPs, respectively. The Fourier transform infrared (FT‐IR) spectroscopy was carried out to verify the formation of condensed aromatic C─N─C networks of mpg‐CN and Pd‐mpg‐CN (Figure S1b, Supporting Information). In terms of mpg‐CN carrier, the band at 810 cm^−1^ belongs to the breathing mode of tri‐s‐triazine units, and the bands from 1200 to 1700 cm^−1^ are related to the stretching vibration of the typical C─N heterocycle. The molecular structure of the mpg‐CN remains unchanged after the introduction of Pd species. Nitrogen adsorption‐desorption isotherms and the corresponding pore size distribution of mpg‐CN and Pd‐mpg‐CN were shown in Figure S2 and Table S1 (Supporting Information). The two samples exhibited type IV isotherms and mesoporous structures. The Brunauer–Emmett–Teller (BET) specific surface area of mpg‐CN is 270 m^2^ g^−1^, which decreases to 176 m^2^ g^−1^ after the deposition of palladium as expected. The morphology of mpg‐CN was confirmed by a series of microscopic analyses including scanning electron microscopy (SEM), transmission electron microscopy (TEM), and HAADF‐STEM (Figures S3–S4, Supporting Information), evidencing the presence of numerous spherical pores (ca. 13 nm) throughout the mpg‐CN carrier, agreeing well with the N_2_ isotherm results. The representative SEM image (**Figure** **1**a) highlights the rough surface structure of Pd‐mpg‐CN, which contributes to its high specific surface area. Further revealed by the HRTEM image, Pd NPs with an average size of 4.4 nm are homogeneously distributed over the surface of Pd‐mpg‐CN (Figure 1b). The inset of Figure 1b shows a single Pd nanoparticle displaying clear lattice fringes with a spacing of 0.23 nm, which corresponds to the (111) plane of the face‐centred cubic (FCC) palladium.

Moreover, the microstructure and compositional distribution of Pd‐mpg‐CN were further investigated by low‐magnification HAADF‐STEM and the corresponding energy dispersive X‐ray spectrometric (EDS) mappings, evidencing the homogeneous distribution of C, N, and Pd throughout the catalyst (Figure 1c). In addition, the aberration‐corrected (AC) HAADF‐STEM analysis of Pd‐mpg‐CN revealed the coexistence of many Pd single atoms (Figure 1d; Figure S5, Supporting Information) positioned in close vicinity to the Pd NPs. To reveal the coordination environments and electronic structure of Pd‐mpg‐CN, X‐ray absorption fine structure (XAFS) measurements were conducted. The Fourier‐transformed (FT) *k* ^2^‐weighted EXAFS analysis showed two peaks at 1.53 and 2.24 Å (Figure 1e), attributed to Pd─N/O and Pd─Pd bonds, respectively. Then, EXAFS fitting was performed to extract the structure parameters of Pd species in Pd‐mpg‐CN. As shown in Figure S6 and Table S2 (Supporting Information), the fitted coordination numbers of Pd─N/O and Pd─Pd were 2.9 ± 0.6 and 1.1 ± 0.6, respectively, with the bond lengths of 1.89 ± 0.01 (Pd─N/O) and 2.70 ± 0.03 Å (Pd─Pd). The fitting result suggests the presence of both Pd single atoms and small‐sized nanoparticles in Pd‐mpg‐CN, corroborating the microscopic analyses. Moreover, the oxidation state of the Pd species in Pd‐mpg‐CN was analyzed by XANES analysis. As shown in Figure 1f, the absorption edge of Pd‐mpg‐CN is located between those of Pd foil and PdO, indicating that the average valence state of Pd in Pd‐mpg‐CN ranges between 0 and +2, the fact confirming the co‐presence of Pd single atoms/ions and Pd(0) nanoparticles. X‐ray photoelectron spectroscopy (XPS) was further conducted to investigate the surface chemical states and the interaction between the Pd species and carbon nitride in Pd‐mpg‐CN, and the survey spectra demonstrate the presence of C, N, O, and Pd in Pd‐mpg‐CN (Figure S7, Supporting Information). Three distinct valence states of Pd at 335.1, 336.5, and 338.3 eV were clearly observed (Figure 1g), which are assigned to Pd^0^, Pd^2+^, and Pd^4+^, respectively. Theoretical modeling of the Pd centres interacting with the mpg‐CN support were performed by density functional theory (DFT/B3LYP or DFT/BP86, with basis 6–31G^*^/LANL2DZ). The optimized structures, in which various oxidation states of Pd have been considered, are given in the (Figure S38, Supporting Information). It was found that the Pd‐N distances (d_Pd‐N_) significantly decreased upon increasing the Pd oxidation state (for Pd^0^, d_Pd‐N_ = 2.48 Å; for Pd^2+^, d_Pd‐N_ = 2.13 Å; for Pd^4+^, d_Pd‐N_ = 2.04 Å). Therefore, both XPS findings and theoretical calculations confirm that the Pd species in Pd‐mpg‐CN possess different coordination environments, in agreement with the EXAFS results. As shown in the donut plot (inset in Figure 1g), the percentages of Pd^0^ (red), Pd^2+^ (blue), and Pd^4+^ (green) in the Pd‐mpg‐CN were determined 47%, 25%, and 28%, respectively (obtained by fitting the peak areas from the deconvoluted XPS spectrum). The C 1*s* spectrum of the Pd‐mpg‐CN remains unchanged after the introduction of the Pd species (Figure S8a, Supporting Information). However, the peaks in the N 1*s* spectrum shift to higher binding energies in the case of Pd‐mpg‐CN (Figure S8b, Supporting Information), which is caused by a decreased electron density and signifies a strong interaction between Pd and nitrogen ligands in the Pd‐mpg‐CN structure.

### 2.2 Photoelectrochemical Properties

**Figure 2:**
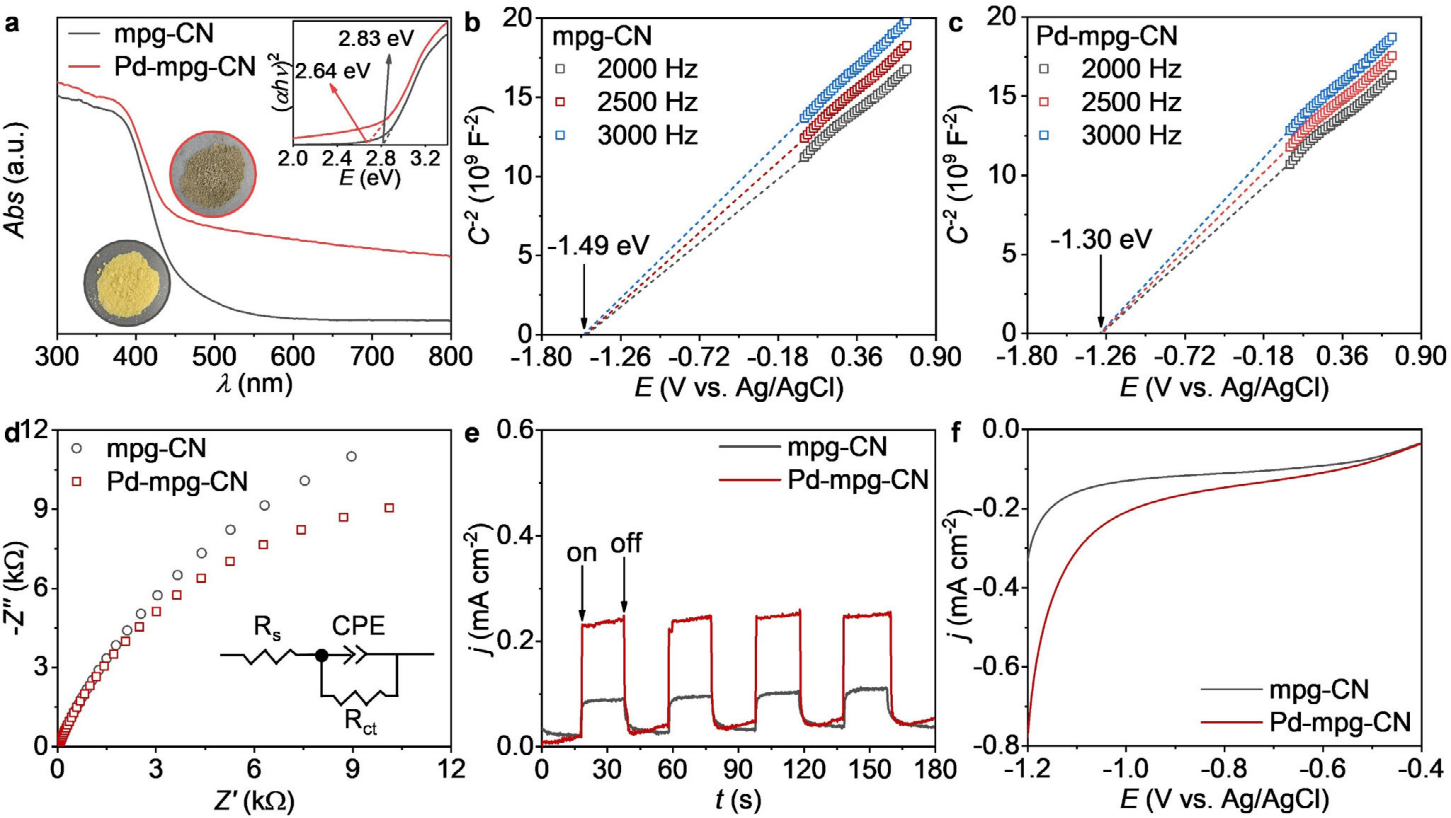
a) UV–vis absorption spectra and Tauc plots (inset) of mpg‐CN and Pd‐mpg‐CN. Mott–Schottky plots of b) mpg‐CN and c) Pd‐mpg‐CN collected at various frequencies. d) Electrochemical impedance spectra, e) transient photocurrent responses, and f) linear sweep voltammetry plots of mpg‐CN and Pd‐mpg‐CN.

As shown in the UV–vis diffuse reflectance spectra (DRS) in **Figure** **2**a, the absorption edge of Pd‐mpg‐CN exhibits an obvious red shift compared to that of mpg‐CN carrier, suggesting that the incorporation of Pd species could significantly enhance the absorption of visible light. The bandgaps (*E*_g_) of mpg‐CN and Pd‐mpg‐CN were determined to be 2.83 and 2.64 eV, respectively (inset in Figure 2a). The smaller *E*_g_ of Pd‐mpg‐CN manifests that the presence of Pd species is conducive to the absorption of visible light. To further study the electronic diagram, the Mott–Schottky (M─S) plots were measured at 2000, 2500, and 3000 Hz to identify the conduction band (CB) positions of the mpg‐CN and Pd‐mpg‐CN (Figure 2b,c; Figure S9, Supporting Information). The as‐prepared materials show a positive slope, which is characteristic of n‐type semiconductors, suggesting that the values of flat band potentials approximately correspond to the CB positions of these samples.^[^ ^32^ ^]^ Therefore, the CB positions of the mpg‐CN and Pd‐mpg‐CN are determined to be −1.49 and −1.30 V (vs Ag/AgCl), corresponding to −0.89 and −0.70 V (vs RHE), respectively. The corresponding band structure alignments of the mpg‐CN and Pd‐mpg‐CN are schematically described in Figure S10 (Supporting Information).

To investigate the charge carrier separation, recombination, and transfer behaviors of mpg‐CN and Pd‐mpg‐CN, electrochemical impedance spectroscopy (EIS) and transient photocurrent response measurements were performed. As shown in Figure 2d, Pd‐mpg‐CN exhibits a smaller arc radius than that of mpg‐CN, manifesting its lower charge–transfer resistance during catalytic reactions. Then, the *I–t* curves reveal that the Pd‐mpg‐CN photoelectrode possesses a much higher transition photocurrent response, which indicates the introduction of Pd species could effectively suppress the recombination of photogenerated electron‐hole pairs (Figure 2e). To study the relation between the reaction kinetic factor and photocatalytic hydrogen evolution over these catalysts, linear scan voltammograms (LSV) were measured (Figure 2f). The Pd‐mpg‐CN exhibits a much higher cathodic current compared to mpg‐CN, regardless of the applied potential, indicating Pd‐mpg‐CN is more favorable for hydrogen evolution than mpg‐CN, which could facilitate the transfer hydrogenation reaction.

### 2.3 Photocatalytic Performance

**Figure 3:**
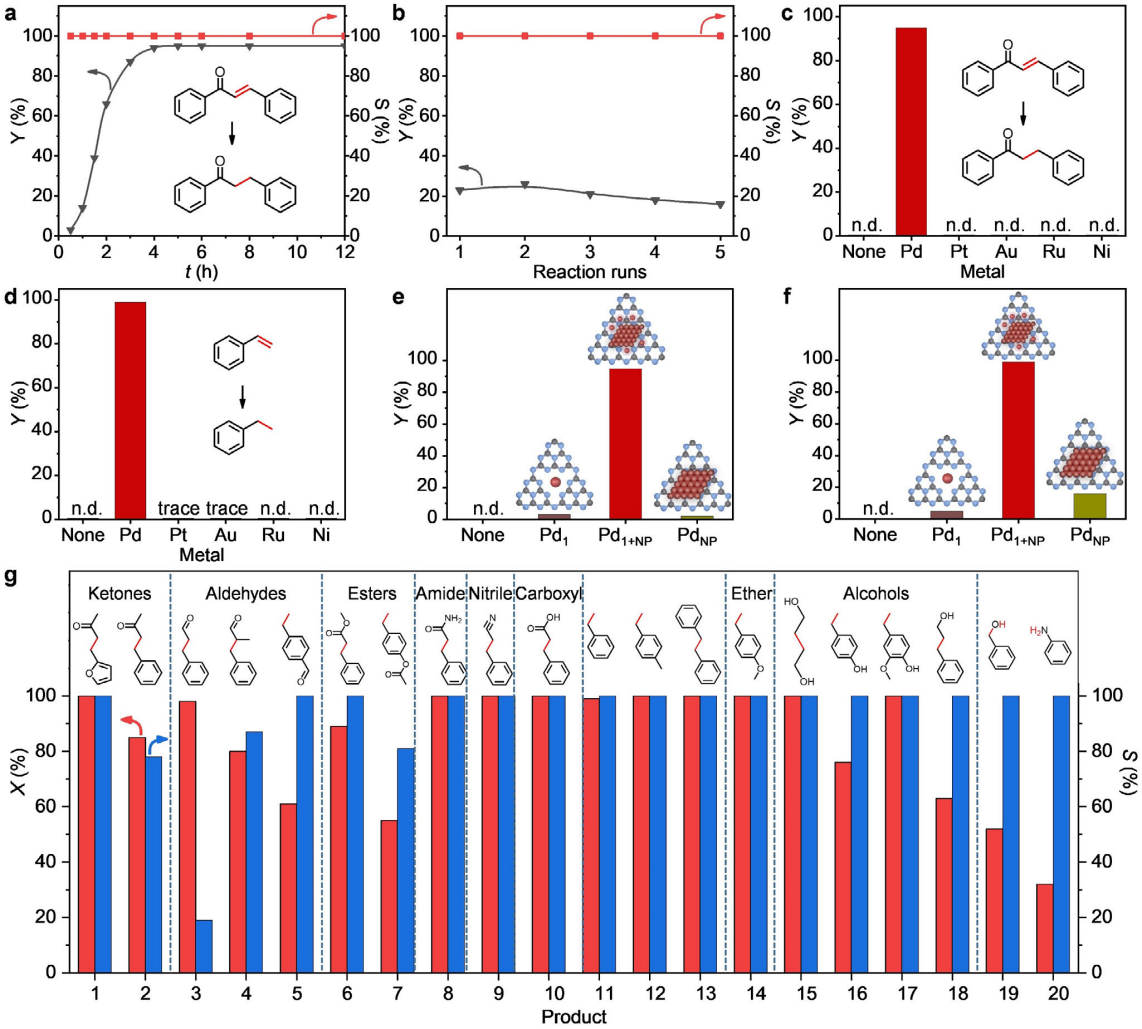
a) The effect of reaction time over Pd‐mpg‐CN for photocatalytic water‐donating selective transfer hydrogenation of chalcone. Reaction conditions: catalyst (10 mg), chalcone (0.1 mmol), ultrapure water (2 ml), 1,4‐dioxane (3 ml), triethylamine (TEA; 0.4 ml), blue light 40 W (λ = 427 nm), reaction temperature (313 K), reaction pressure (1 bar), N_2_ atmosphere. b) Reusability testing of Pd‐mpg‐CN. The stability test was performed under the same photocatalytic reaction conditions as in (a) except for a reaction time of 1.5 h. Photocatalytic performance using c) chalcone or d) styrene as a substrate over different metal catalysts. Photocatalytic performance using e) chalcone or f) styrene as a substrate over different palladium configurations. n.d. = not detected. Reaction conditions as indicated in (a) except for a reaction time of 4 h. g) Reaction scope of Pd‐mpg‐CN for photocatalytic water‐donating selective transfer hydrogenation. The scope test was performed under the same photocatalytic reaction conditions as in (a) except for different reaction times: 4 h (entries 2–4, 8–16, and 19–20) and 10 h (entries 1, 5–7, and 17–18). Red bars (*X*%) stand for conversion and blue bars (*S*%) for selectivity.

The photocatalytic performance of the prepared catalysts was first evaluated in the water‐donating selective transfer hydrogenation of chalcone under visible light irradiation and ambient conditions. Through screening (Figures S11 and S12, Supporting Information), the 2.9 wt.% Pd‐loading photocatalyst and triethylamine (TEA) were identified as the optimal catalyst and hole scavenger, respectively. Control experiments revealed that the absence of the corresponding ketone compound (1,3‐diphenylpropan‐1‐one) was observed when the experiments were performed in the dark, or without water or Pd‐mpg‐CN or TEA, suggesting that these conditions are essential (Table S3, Supporting Information, entries 1–5). The effect of reaction time was then studied at 313 K (**Figure** **3**a). With the reaction time within 4 h, the conversion of chalcone increased progressively from 8% (0.5 h) to 95%, and the selectivity for 1,3‐diphenylpropan‐1‐one remained >99% throughout the reaction. Meanwhile, the as‐prepared Pd‐mpg‐CN exhibited satisfactory stability, despite that a slight decay was observed in the conversion after five consecutive reaction runs (Figure 3b), probably due to the partial aggregation of Pd NPs (Figures S13 and S14, Supporting Information). The XPS analysis of the used Pd‐mpg‐CN revealed that the content of zero‐valent Pd remains unchanged (Figure S15, Supporting Information), while a portion of the Pd^2+^ was converted to Pd^4+^ species, which could be attributed to a two‐electron oxidation process during the reaction (vide infra).

To study the metal‐dependent catalysis for the photocatalytic water‐donating selective transfer hydrogenation of chalcone, four additional mpg‐CN‐supported noble metal catalysts including Pt‐mpg‐CN, Au‐mpg‐CN, Ru‐mpg‐CN, and Ni‐mpg‐CN were prepared with the same method as Pd‐mpg‐CN (Figures S16–S18 and Table S1, Supporting Information) for benchmarking. Surprisingly, all these noble metal catalysts were found inert for this type of reaction (Figure 3c). Intentionally, we traced the generation and consumption of the hydrogen protons (Figure S19, Supporting Information), which is an intermediate for both the transfer hydrogenation and hydrogen evolution reactions. When the substrate was absent, a large amount of hydrogen was generated over the Pd‐mpg‐CN, but these hydrogen protons participated in the hydrogenation reaction after the substrate was added. Multivalence‐state Pd species exhibit a unique hydrogen proton adsorption feature, which enables optimal balance between H* adsorption from water splitting and subsequent desorption for transfer hydrogenation. However, the Pt‐mpg‐CN prepared by the same method as Pd‐mpg‐CN exhibited only a monovalent state (Pt^2+^, Figure S18, Supporting Information), which may lead to the inactivation of the Pt‐mpg‐CN catalyst. In terms of other metal‐based catalysts (i.e., Au, Ru, Ni), either the hydrogen evolution activity is weak or the hydrogenation activity is feeble, ultimately leading to inactive photocatalytic transfer hydrogenation. To gain more quantitative insights, the apparent quantum efficiencies (AQEs) for transfer hydrogenation and hydrogen evolution were calculated, which were ≈0.9% and 0.006%, respectively. This indicates explicitly that the majority (>99%) of the photogenerated hydrogen protons had been utilized for the transfer hydrogenation reaction in the case of Pd‐mpg‐CN. In addition, the action spectra (Figure S20, Supporting Information) displayed the optical absorption spectra of Pd‐mpg‐CN under irradiation with a 300 W Xe lamp equipped with different bandpass filters (365–700 nm), showing that the wavelength‐dependent trend of AQE aligns with variations in light absorption. We further employed the most common styrene as a model substrate, which showed the same catalytic behavior as chalcone, i.e., only the Pd‐mpg‐CN demonstrated reasonable performance (yield of ethylbenzene formation, 99%), while the other metal‐based catalysts were either inactive or with trace yields (Figure 3d).

To further explore the structure‐performance relationships in this type of reaction, a Pd nanoparticle‐only catalyst (Pd_NP_‐mpg‐CN) (Figures S21 and S22, Supporting Information) and a single‐atom‐only catalyst (Pd_1_‐mpg‐CN) (Figures S23–S25 and Table S2, Supporting Information) were prepared intentionally to see the effects of Pd nanoparticles and Pd single atoms if applied separately (without any possibility of the synergistic action). Interestingly, both the Pd_1_‐mpg‐CN and Pd_NP_‐mpg‐CN presented trace activity for the selective transfer hydrogenation of chalcone under light irradiation (Figure 3e). Moreover, an additional Pd nanoparticle catalyst was prepared via high‐temperature hydrogen reduction (Pd_NP_‐mpg‐CN‐1), which also exhibited low activity (a yield of 7.8%, Table S3, Supporting Information, entry 6). Similar catalytic behavior was observed for the photocatalytic transfer hydrogenation of styrene (Figure 3f), in which Pd‐mpg‐CN (containing both nanoparticles and single atoms) exhibited excellent catalytic activity (*Y*_ethylbenzene_ = 99%), while Pd_1_‐mpg‐CN (*Y*_ethylbenzene_ = 5%) and Pd_NP_‐mpg‐CN (*Y*_ethylbenzene_ = 16%) showed significantly lower performance. Thus, it is clear that the catalytic effectiveness of Pd‐mpg‐CN is due to the formation of unique synergistic sites by combining Pd^2+/4+^ single atoms and Pd(0) nanoparticles. This was further proved by detailed electron paramagnetic resonance analyses (as discussed later in the “Mechanism analysis”).

The versatility of the optimized protocol was assessed by using a variety of substrates with different functional groups, including ketones, aldehydes, alcohols, esters, etc. As shown in Figure 3g, Pd‐mpg‐CN exhibited high catalytic activity with >99% conversion and >99% selectivity toward furfurylideneacetone (entry 1). The benzalacetone was also hydrogenated to the desired ketone in good conversion (85%) and selectivity (78%) (entry 2). For cinnamaldehyde, nearly full conversion was achieved but the benzenepropanal was produced with 19% of selectivity (entry 3), which could be due to the Aldol condensation under alkaline conditions. Interestingly, *α*‐methylcinnamaldehyde without *α*‐H could achieve 80% conversion and 87% selectivity without the condensation reaction (entry 4). The 4‐vinylbenzaldehyde was reduced to 4‐ethylbenzaldehyde with 61% conversion and >99% selectivity (entry 5). Besides, the conversion of methyl cinnamate reached 89% with a methyl 3‐phenylpropanoate selectivity exceeding 99% (entry 6). The excessive hydrogenation of 4‐acetoxystyrene to 4‐hydroxystyrene and 4‐ethylphenol leads to 81% selectivity with 55% conversion (entry 7). Next, the industrially important styrene and its derivatives (i.e., amide, nitrile, carboxyl, ether, and alcohol) were further investigated (entries 8‐18), in which the Pd‐mpg‐CN exhibited impressive activity for the selective photocatalytic transfer hydrogenation of C═C double bonds in quantitative yields. Furthermore, the hydrogenation of benzaldehyde (a yield of 52%) and nitrobenzene (a yield of 32%) demonstrated that Pd‐mpg‐CN exhibited moderate catalytic activity across distinct double‐bond systems. These data confirm the unique practical potential of the developed mixed‐valence palladium system acting as the first universal photocatalyst for the transfer hydrogenation of a broad scale of organic compounds with water as a proton source. Moreover, the possibility of the Pd‐mpg‐CN for large‐scale applications was proved on a gram scale, achieving a 99% yield of 1,3‐diphenylpropan‐1‐one (Figure S26, Supporting Information).

### 2.4 Mechanism Analysis

Motivated by the unique structure of mixed‐valence state (0/2+/4+) palladium catalyst and its impressive photocatalytic performance, we made a significant effort to address the mechanistic action beyond that. This motivation was further strengthened by the negligible activities expressed by other noble metal‐based catalysts, which were prepared by the same synthetic procedure, and by the very low activity of Pd NPs or Pd SAs if applied separately (without any possibility of the synergistic action).

**Figure 4:**
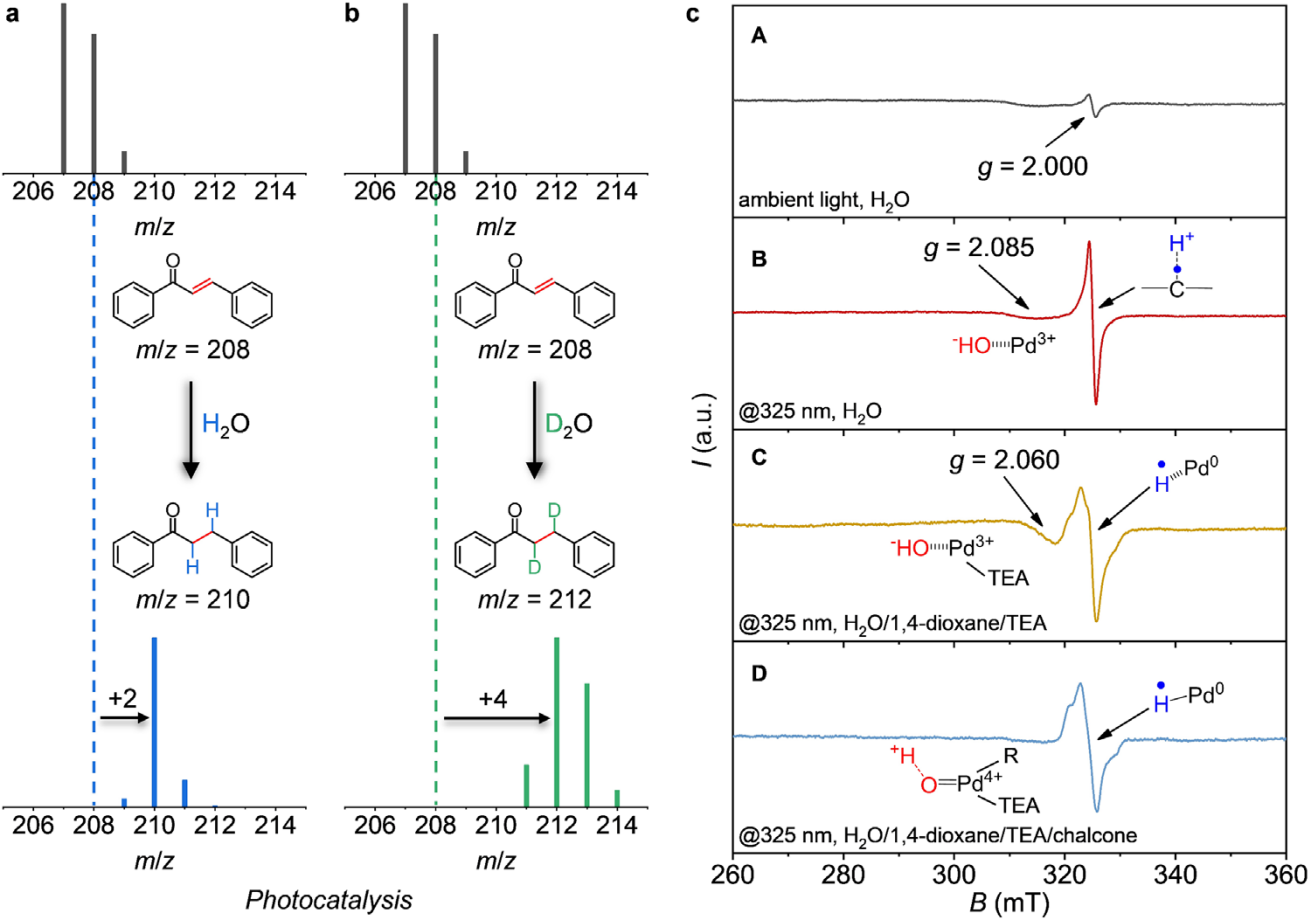
Mass spectra of the liquid product from the photocatalytic water‐donating selective transfer hydrogenation of chalcone in the presence of Pd‐mpg‐CN in a) H_2_O and b) D_2_O, respectively. c) CW X‐band (9.08 GHz) EPR spectra of Pd‐mpg‐CN recorded at *T* = 85 K in A) water under ambient light irradiation, B) water with in situ 325 nm irradiation, C) water/1,4‐dioxane/TEA mixture with in situ 325 nm irradiation, and D) water/1,4‐dioxane/TEA/chalcone with in situ 325 nm irradiation.

The proton source of photocatalytic transfer hydrogenation from water splitting was first confirmed by isotope‐labeling experiments. As shown in **Figure** **4**a, a molecular ion peak of the 1,3‐diphenylpropan‐1‐one at *m*/*z* = 210 (Figure S27, Supporting Information) of the gas chromatography‐mass spectrometry (GC‐MS) was observed after the photocatalytic transfer hydrogenation in H_2_O with chalcone (*m*/*z* = 208, Figure S28, Supporting Information) as the substrate. Instead, a deuterated 1,3‐diphenylpropan‐1‐one product (*m*/*z* = 212, Figure S29, Supporting Information) was identified when the reaction was performed in D_2_O (Figure 4b), evidencing the direct water‐donating protonation of chalcone.

To unveil the presence of spin‐containing photoexcited states (electron/hole) in the Pd‐mpg‐CN system, gain clear evidence for the generation of H^•^ intermediates from water, and obtain a detailed picture of the catalytic steps involved in the transfer hydrogenation reaction, the EPR technique was employed.^[^ ^33^ ^]^ Under ambient light irradiation (Figure 4c‐A; Figure S30, Supporting Information), when the catalyst in pure water was fast cooled down from room temperature to 85 K, the EPR spectrum showed a weak *S* = ½ signal that became highly enhanced under in situ light irradiation (Figure 4c‐B; S30, Supporting Information). This isotropic EPR resonance signal at *g* = 2.000 is associated with the effective generation of photoexcited electrons localized on C‐containing centers of the mpg‐CN backbone. On the other hand, the photogenerated holes do not give any clear resonance signal, thus h^+^ are highly delocalized onto the mpg‐CN support. Nevertheless, it is also observed the presence of a broad and weak Pd^3+^ resonance signal (in the region where *g_eff_* > *g*_e_ = 2.0023), which indicates that fraction of photogenerated h^+^ were capable to successfully oxidize some Pd^2+^ centers (Pd^2+^ + h^+^ → Pd^3+^). When 1,4‐dioxane was added to water, the resulting low‐temperature EPR spectra (Figure S31, Supporting Information), recorded at ambient light and under light irradiation, did not change compared to those seen earlier in neat water (Figure S30, Supporting Information). Therefore, the nature and electronic characteristics of the photogenerated spins (e^−^/h^+^) were not altered by the presence of the organic solvent (Figure S32, Supporting Information). Addition of 1,4‐dioxane/TEA to the water solution significantly changed the EPR fingerprints of the catalytic system under in situ light irradiation (Figure 4c‐C; Figure S33, Supporting Information), and the signal ≈g = 2.000 became highly anisotropic (g_x_ = 2.018, g_y_ = 2.002, g_z_ = 1.993). This is consistent with the formation of H• radical species that interact with the Pd0 nanoparticles (H•…Pd), showing resolved nuclear hyperfine term (HFI, 1H) of A_x_ = 2.30 mT, A_y_ = 2.10 mT, A_z_ = 1.46 mT, as obtained from simulation of the EPR resonance envelope obtained by perturbation theory (Figure S33, Supporting Information). Moreover, the electron spin moment of the formed Pd^3+^ sites (Pd^2+^ + h^+^ → Pd^3+^) coupled with one ^14^N nuclei from interacting TEA molecule, giving hyperfine term (*A*_N_) along the *g*‐parallel component of 17.8 mT and Pd axial anisotropy (*g*_//_ = 2.315 and *g*_⊥_ = 2.060) (Figures S40 and S41, Supporting Information). Therefore, during the photoexcitation process and generation of polaron charges (e^−^/h^+^), the emergence of nuclear hyperfine interactions (HFI) involving Pd single atoms and TEA molecules indicates that TEA acted as electronic modulator, enhancing localization and better interaction of h^+^ with single atom Pd^2+^ sites whilst facilitating the delivery of e^−^ to H^+^.^[^ ^34^, ^35^ ^]^ The EPR spectrum of the Pd‐mpg‐CN recorded in water/1,4‐dioxane mixture in the presence of chalcone only showed a weak anisotropic resonance feature at *g* = 2.000 (Figure S34, Supporting Information), which was found almost identical to that observed in water (Figure S30, Supporting Information) or water/1,4‐dioxane mixture (Figure S31, Supporting Information) recorded without substrate. When the substrate was added to the complete solution of reactants (water/1,4‐dioxane/TEA), the EPR signal, recorded at 85 K, and associated with the Pd^3+^ sites disappeared (Figure 4c,D; Figure S35, Supporting Information), indicating that a two‐electron oxidation process occurred (Pd^2+^ + 2h^+^ → Pd^4+^)(Figure S42, Supporting Information). Furthermore, the process was fast when the substrate was present, manifesting that the substrate was bound or strongly interacting to the single‐atom Pd sites. Then, the two‐electron reduction (H^+^ + e^−^ → H^•^ + e^−^ → H^−^) was slower because did require the occurrence of H^−^ spillover from the hydride bound to the Pd nanoparticles, and the subsequent H^−^ transfer to a nearby Pd single atom center where the substrate was located. For comparison, and in stark contrast, the recorded EPR fingerprints for the inactive catalyst, Ni‐mpg‐CN (Figure 3c), revealed that the oxidation state (+2) of Ni did not change in presence of the chalcone substrate and under light irradiation. These properties underline that Ni‐mpg‐CN has poor proclivity to interact with photogenerated h^+^, thus, to effectively sustain transfer hydrogenation reactions to the chalcone molecule, using water as proton source (Figure S37, Supporting Information).

Finally, the EPR results for Pd‐mpg‐CN obtained by replacing H_2_O with D_2_O validated further that the proton source that drives the hydrogenation reaction originates from water, and not from chemical reactions of TEA molecules (Figure S36, Supporting Information).

**Figure 5:**
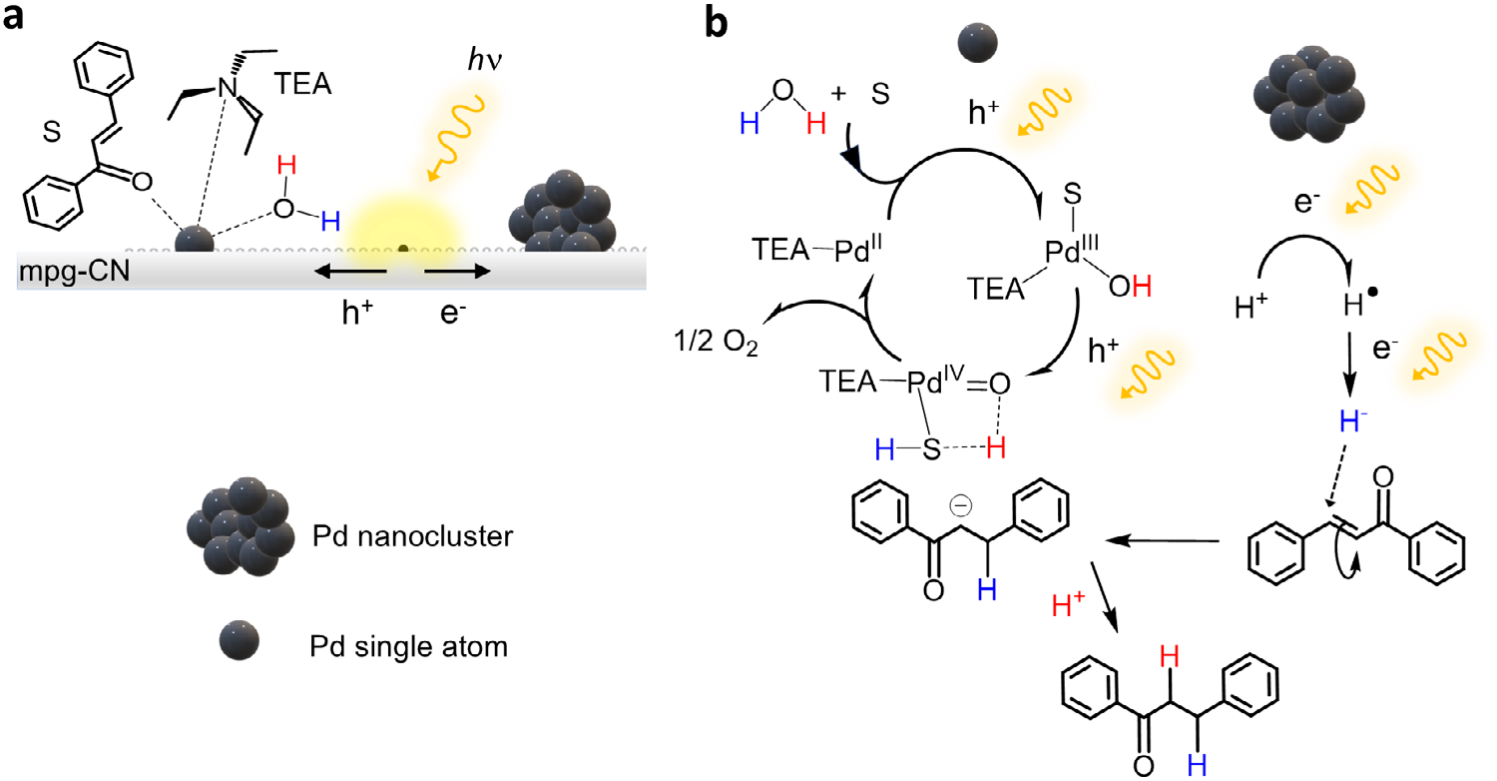
The pathway for photocatalytic water‐donating selective transfer hydrogenation of chalcone in the presence of Pd‐mpg‐CN. Under light irradiation (panel **a**), electron and hole species (e^−^/h^+^) are generated near the spin‐containing defects in the mpg‐CN support. These polaron species moves toward different sites of the Pd catalyst, hence preventing fast recombination. On panel **b** the single atom Pd centers become active for substrate binding and substrate hydrogenation. The Pd nanoparticles act as sites competent for H^+^ binding and hydride (H^−^) formation. TEA functions as electronic modulator. It aids the transfer of e^−^ to H^+^ and localization of h^+^ to single atom Pd via hyperfine interactions.

Based on the above findings, the catalytic pathway for the transfer hydrogenation reaction carried out by Pd‐mpg‐CN is shown in **Figure** **5**. Overall, the photogenerated electrons and holes act in a synergic manner: i) the spin‐containing defects embedded in the carbon nitride support (mpg‐CN) together with Pd single atoms and nanoparticles do provide the anisotropic environment that allow slow recombination of the photogenerated e^−^/h^+^ species, facilitating e^−^/h^+^ migration to different regions of the catalysts,^[^ ^36^ ^]^ ii) the Pd^0^ nanoparticles work as sites competent for H^+^ to H^−^ reduction; iii) the formed H^−^ migrate toward Pd single atoms where the substrate was located via hydrogen spillover followed by hydrogenation reaction. Moreover, TEA acts an electronic modulator that facilitates polarization of the photoexcited electron‐hole pairs, directing electrons to the Pd^0^ nanoparticles and contributing to the localization of the holes toward the Pd^2+^ single‐atom sites due to the binding tendency of TEA to these Pd atoms.

## 3 Conclusion

In conclusion, we have developed an efficient photocatalytic selective transfer hydrogenation technology for conversion of unsaturated C═C bonds in organic compounds via the synergy of Pd single atom centers and Pd nanoparticles on carbon nitride using water as a proton source in the absence of any protective reagents or additives. The developed system, containing Pd^2+^, Pd^4+^ single atoms, and Pd^0^ nanoparticles, acts as a universal catalyst enabling to convert a broad portfolio of organic substrates with unsaturated C═C bonds with very high yield and selectivity. For example, remarkable conversion (95%) and selectivity (>99%) was achieved for transfer hydrogenation of chalcone to 1,3‐diphenylpropan‐1‐one. In addition, the Pd‐mpg‐CN photocatalyst presented excellent catalytic activity toward the selective C═C bond hydrogenation in the presence of various functional groups such as ketones, aldehydes, alcohols, esters, etc. The isotope‐labeling experiments and EPR analysis demonstrated that the proton source for transfer hydrogenation originates from water. Furthermore, the *operando* light‐induced EPR confirmed the synergistic action between Pd single atoms and nanoparticles, in which the H^−^ produced by Pd NPs migrate to Pd single atoms where the substrate was located through hydrogen spillover, resulting in the transfer hydrogenation of substrate. The developed catalytic system possesses several advantages over current hydrogenation technologies, mainly the use of light irradiation as a driving force for chemical processes and water as the proton source to replace flammable H_2_. Moreover, the synergy between Pd single atom centers and nanoparticles allows to maximize atom utilization for efficient production of high value‐added organics with the applicability for a broad portfolio of substrates. We believe, this new concept of the redox cooperativity of various Pd centers would facilitate the implementation of photocatalytic transfer hydrogenation process with water as a proton source in the real chemical and industrial practice.

## 4 Experimental Section

Experimental details, including materials, characterizations, photoelectrochemical measurements, and calculation of apparent quantum efficiency, are provided in the Supporting Information.

### Synthesis of Mpg‐CN

Cyanamide (5 g) was added to an aqueous dispersion of SiO_2_ nanoparticles (16.25 g, 40 wt.% solids, 12 nm average diameter), then the mixture was stirred for 6 h at 333 K to evaporate absolutely the water. The resulting solids were ground thoroughly and heated to 823 K (2.3 K min^−1^ ramp rate) for 4 h under an air atmosphere. After it was cooled to room temperature, the product was treated with 4 M NH_4_HF_2_ solution (150 mL) for 48 h to remove SiO_2_ nanoparticles. The carrier was then centrifuged and washed completely with deionized water and ethanol. Finally, the solids were dried at 343 K overnight.

### Synthesis of Metal‐Mpg‐CN

The mpg‐CN (0.5 g) was added to deionized water (40 mL) under sonication for 1 h. Then, the metal precursor (PdCl_2_, H_2_PtCl_6_·*x*H_2_O, HAuCl_4_·3H_2_O, RuCl_3_, or NiCl_2_, 3.0 wt.% metal relative to the carrier) was added and stirred overnight. The slurry was further stirred for 5 h at 333 K. The solids were collected by centrifuging, washed with deionized water and ethanol completely, and dried at 333 K overnight. Finally, it was treated at 473 K with a rate of 5 K min^−1^ for 5 h under a 10% H_2_/Ar atmosphere. Moreover, samples with 1.5 and 4.5 wt.% Pd loading were obtained with the addition of PdCl_2_ of 0.0127 and 0.0258 g.

### Synthesis of Pd_1_‐Mpg‐CN

The mpg‐CN (0.5 g) was dispersed in water (40 mL) under sonication for 1 h. Then, an aqueous solution of 10 wt.% Pd(NH_3_)_4_(NO_3_)_2_ (0.0684 mL) was added, respectively, then stirred overnight. Afterward, the above solutions were stirred for 6 h at 333 K. The desired products were collected by centrifuging, washed with deionized water and ethanol, and dried at 343 K overnight. Finally, the solids were treated at 573 K (5 K min^−1^ ramp rate) for 5 h under a nitrogen flow.

### Synthesis of Pd_NP_‐Mpg‐CN

The Pd_NP_‐mpg‐CN was prepared by sodium borohydride reduction method. Specifically, an aqueous solution of PdCl_2_ was added to deionized water. Then, a fresh NaBH_4_ solution was added to the above solution and stirred for 24 h. Next, mpg‐CN was dispersed to the above solutions, sonicated for 1 h, and stirred overnight. Finally, the product was collected by centrifuging and washing with deionized water and ethanol and was dried at 343 K overnight.

### Photocatalytic Test

The photocatalytic water‐donating selective transfer hydrogenation experiments were performed using a 40 W blue LED (PR160L‐427 nm). In brief, the catalyst (10 mg) was dispersed in ultrapure water (2 mL), followed by sonication for 5 min. Subsequently, 1,4‐dioxane (3 mL), chalcone (0.1 mmol), and TEA (0.4 mL) were added, then the reactor was sealed with purification of N_2_. Afterward, it was irradiated with an LED module from a fixed distance of 10 cm, with an irradiation area of ≈6 cm^2^. The irradiance of the LED lamp was 70.4 mW∙cm^−2^. After the reaction, the suspension was centrifuged to remove the solid catalyst and then analyzed by GC‐MS (Shimadzu QP2020 NX) using acetophenone as an internal standard. For the stability test, the catalyst after the reaction was directly reused in the next reaction cycle.

### Statistics

In the hydrogen evolution experiment, 3 experiments were performed at least in duplicate. The XRD and FTIR data were normalized before plotting. The statistical analysis of palladium nanoparticles in the electron microscopy images was performed using Nano Measurer software, with a minimum of 120 particles selected per sample. The percentages of Pd^0^, Pd^2+^, and Pd^4+^ species were determined by deconvoluting the Pd 3*d* XPS spectra and integrating the peak areas using Advantage software.

## Conflict of Interest

The authors declare no conflict of interest.

## Supporting information



Supporting Information

## Data Availability

The data that support the findings of this study are available from the corresponding author upon reasonable request and at https://doi.org/10.5281/zenodo.15405350.
