# Supplementary material for: Atomic Scale Engineering of Multivalence‐State Palladium Photocatalyst for Transfer Hydrogenation with Water as a Proton Source
Source: Adv Mater. 2025 May 22;37(32):2504108. doi: 10.1002/adma.202504108 (PMC12355530; doi:10.1002/adma.202504108)
Supplement: Supplementary file 1 — Supporting Information [file ADMA-37-2504108-s001.pdf]

ADVANCED MATERIALS

Supporting Information

for *Adv. Mater.*, DOI 10.1002/adma.202504108

Atomic Scale Engineering of Multivalence-State Palladium Photocatalyst for Transfer Hydrogenation with Water as a Proton Source

En Zhao, Wenjing Kong, Giorgio Zoppellaro, Yue Yang, Bing Nan, Lina Li, Wengjun Zhang, Zhaohui Chen, Aristides Bakandritsos, Zhu-Jun Wang, Matthias Beller, Radek Zbořil* and Zupeng Chen**

Supporting Information

Atomic Scale Engineering of Multivalence-State Palladium Photocatalyst for Transfer Hydrogenation with Water as a Proton Source

En Zhao,[a] Wenjing Kong,[a] Giorgio Zoppellaro[b,c] Yue Yang,[d] Bing Nan,[e] Lina Li,[e] Wengjun Zhang,[a] Zhaohui Chen,[a] Aristides Bakandritsos,[b,c] Zhu-Jun Wang,[d] Matthias Beller,[f] Radek Zbořil*[b,c] and Zupeng Chen*[a]*

[a]Jiangsu Co-Innovation Center of Efficient Processing and Utilization of Forest Resources, International Innovation Center for Forest Chemicals and Materials, Nanjing Forestry University, Longpan Road 159, Nanjing 210037, China

E-mail: czp@njfu.edu.cn

[b]Regional Centre of Advanced Technologies and Materials, Czech Advanced Technology and Research Institute (CATRIN), Palacký University Olomouc, Šlechtitelů 27, Olomouc 783 71, Czech Republic

E-mail: radek.zboril@upol.cz

[c]Nanotechnology Centre, CEET, VSB-Technical University of Ostrava, 17. listopadu 2172/15, Ostrava-Poruba 708 00, Czech Republic

[d]School of Physical Science and Technology, Shanghai Tech University, Shanghai 201210, People's Republic of China

[e]Shanghai Synchrotron Radiation Facility, Shanghai Advanced Research Institute, Zhangheng Road 293, Shanghai 201204, China

[f]Leibniz-Institute for Catalysis, Albert-Einstein-Straβe 29a, Rostock 18059, Germany

Experimental Section

Materials

Cyanamide (95%, Macklin), LUDOX® HS-40 colloidal silica (40 wt.% suspension in H_2O, Sigma-Aldrich), ammonium hydrogen difluoride (98%, Sinopharm), palladium chloride (59 wt.% Pd, Sinopharm), chloroplatinic acid hydrate (99.995%, Aladdin), gold chloride trihydrate (99.9%, Aladdin), ruthenium trichloride (99.6%, Sinopharm), nickel chloride (99%, Macklin), 1,4-dioxane (99.5%, Sinopharm), triethylamine (99%, Aladdin), acetophenone (99.5%, Macklin), chalcone (98%, Aladdin), 4-(2-furyl)-3-buten-2-one (98%, Aladdin), benzalacetone (99.5%, Macklin), sodium sulfate (99%, Sinopharm), Nafion (5 wt.%, Sigma-Aldrich). All chemicals were used as received without further purification. Theoretical calculations (geometry optimization) of models for the Pd single atom centers interacting with the *mpg*-CN support were performed by density functional theory (DFT) in the gas phase using B3LYP or BP86 functional with the Euler-Maclaurin-Lebedev grid (70, 302) and basis set 6-31G* for C,H,N and effective core-potential (ECP, LANL2DZ) for Pd, as implemented in the Spartan 10 (ver. 1.1.0, Wavefunction Inc., Irvine, CA 92612) computational package. The SCF convergence and gradient convergence were set to 10^{-7} au and <0.0003, respectively. To probe the interactions of the substrate (chalcone molecule), TEA, and substrate plus TEA molecules with single atom Pd centers, and to screen the possible conformational arrangements of the interacting systems, we used a combination of Merk Molecular Force Field[a] (MMFF94)/Monte Carlo (MC) methods. The method (MMFF94/MC) employs simulated "annealing" to generate a set of accessible conformations of the molecules within an energy window. The initial temperature for the Monte Carlo (MC)/Simulated-Annealing algorithm was set to T = 5000 K. Restricted searches were applied, with E_{max} = 40 kJ mol^{-1}, and a pool of 3000 conformers screened. The best scored conformers were then selected and refined by geometry optimization using the PM3(tm) semiempirical method,[b] as implemented in the Spartan 10 (ver. 1.1.0) computational suite.

a. T. A. Halgren. Merck molecular force field. I. Basis, form, scope, parameterization, and performance of MMFF94. *J. Comput. Chem.* **1996**, 490-519.

b. (a) A. S. Christensen, T. Kubař, Q. Cui, and M. Elstner. Semiempirical Quantum Mechanical Methods for Noncovalent Interactions for Chemical and Biochemical Applications. *Chem. Rev.* **2016**, 116, 5301-5337. (b) T. R. Cundari, J. Deng. PM3(tm) Analysis of Transition-Metal Complexes. *J. Chem. Inf. Comput. Sci.* **1999**, 39, 2, 376–381.

Characterizations

Powder X-ray diffraction (XRD) patterns were monitored at an Ultima IV X-ray diffractometer using Cu Kα radiation (λ = 1.541841 Å). Fourier-transformed infrared (FT-IR) spectra were obtained using KBr pellets with a VERTEX 80V spectrometer. Brunauer-Emmett-Teller (BET) specific surface area was determined by nitrogen adsorption-desorption isotherm measurements at 77 K (ASAP 2460). X-ray photoelectron spectroscopy (XPS) measurements were performed at a Thermo ESCALAB 250XI with an Al anode (Al-Kα = 1486.6 eV), in which binding energies were corrected by reference to the C 1s peak at 284.8 eV. A JEM 2100F TEM/STEM recorded the transmission electron microscopy (TEM), the high angle annular dark-field scanning transmission electron microscopy (HAADF-STEM), and energy-dispersive spectroscopy (EDS) images. Scanning electron microscopy (SEM) images were acquired on a Hitachi S-4800 instrument. Ultraviolet-visible diffuse reflectance spectra (UV-vis DRS) were recorded by a Lambda 950 spectrophotometer with $BaSO_4$ as the reflectance standard. The mass fraction of Pd in the as-prepared catalysts was determined by an inductively coupled plasma-optical emission spectrometry (ICP-OES) using an Inductively Coupled Plasma Optical Emission Spectrometer (Varian ICP-OES 720). CW X-band Electron paramagnetic resonance (EPR) spectra were recorded at the temperature of 85 K using a JEOL JES-X-320 spectrometer equipped with variable temperature control ES 13060 DVT5 apparatus. The cavity Q quality factor was kept above 6000 in all measurements. Highly pure quartz tubes were employed (Suprasil, Wilmad, ≤ 0.5 OD), and accuracy on *g*-values was obtained against a Mn^{2+}/MgO standard (JEOL standard). The microwave power was set to 1.00 mW in all measurements to avoid saturation effects. For the in-operando Light-induced Electron Paramagnetic Resonance experiments (LEPR), a HeCd laser operating at 325 nm (200 mW) was employed by directly fitting, in which an optical wire the light source into the dedicated optical window of the EPR resonator.

Photoelectrochemical measurements

Electrochemical measurements were examined by a CHI 760E electrochemical workstation (Shanghai Chenhua, China) with a standard three-electrode cell consisting of the prepared electrode as the working electrode, platinum foil as the counter electrode, and Ag/AgCl (saturated KCl) as a reference electrode. The three-electrodes were immersed in a sodium sulfate electrolyte solution (0.2 M). For the preparation of the working electrode, the catalyst (5 mg) and Nafion solution (0.05 mL) were added to an aqueous solution (0.45 mL) under sonication for 3 h. The above slurry (0.015 mL) was dropped onto a fluorine-tin oxide (FTO) glass electrode and dried at 343 K. The coating thickness of the catalysts in the cross-section of the

FTO in SEM images (Figure S18) is almost the same. The electrochemical impedance spectroscopies (EIS) were performed at 0 V (vs. Ag/AgCl) with an amplitude of 5 mV and a frequency range from 10^5 to 10^{-1} Hz. The transient photocurrent responses were obtained using a 40 W blue LED at 0 V (vs. Ag/AgCl, pH = 6.8) bias potential. The linear sweep voltammetry (LSV) experiments were recorded at a potential window ranging from -1.2 to 0.2 V with a scan rate of 10 mV/s. The Mott-Schottky curves were determined at a potential window ranging from -0.7 to 0.7 V, in which the perturbation signal was an AC voltage magnitude of 5 mV at different frequencies (2000, 2500, and 3000 Hz).

Calculation of apparent quantum efficiency (AQE)

The apparent quantum efficiencies (AQEs) for the photocatalytic water-donating selectivity transfer hydrogenation and hydrogen evolution were measured under the irradiation of 427 nm LED and the reaction conditions (0.1 mmol chalcone, 2 ml ultrapure water, 3 ml 1,4-dioxane, 0.4 ml TEA, 40 W blue light (λ = 427 nm), 313 K, 1 bar, 8 h, and N_2 atmosphere). The irradiation area was controlled to about 6 cm^2. the irradiance was measured to be 70.4 $mW \cdot cm^{-2}$ by a CEL-NP2000 photoradiometer (Beijing CEAULIGHT). The AQEs were calculated as follows:

$$\mathrm{AQE} = \frac{N_e}{N_p} \times 100\% = \frac{2 \times M \times N_A \times h \times c}{A \times I \times t \times \lambda} \times 100\%$$

Where N_p is the total number of incident photons, N_e is the total number of reactive electrons, M is the mole number of H_2 molecules or ethylbenzene molecules, N_A is the Avogadro constant, h is the Planck constant, c is the speed of light, A is the irradiation area, I is the intensity of irradiation light, t is the photoreaction time, λ is the wavenumber of indecent light.

Supplementary Figures

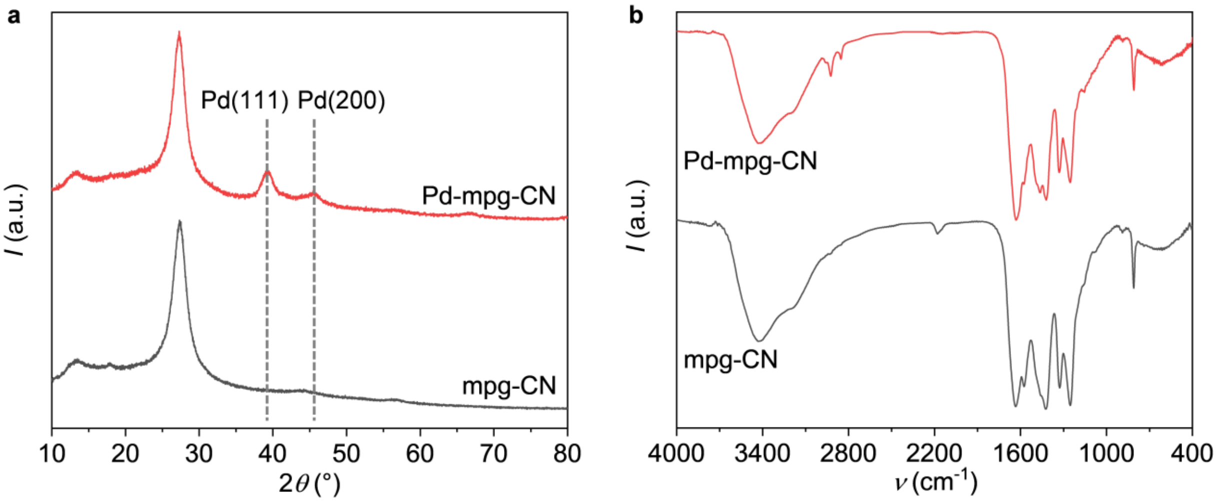

Figure S1. (a) XRD patterns and (b) FT-IR spectra of mpg-CN and Pd-mpg-CN.

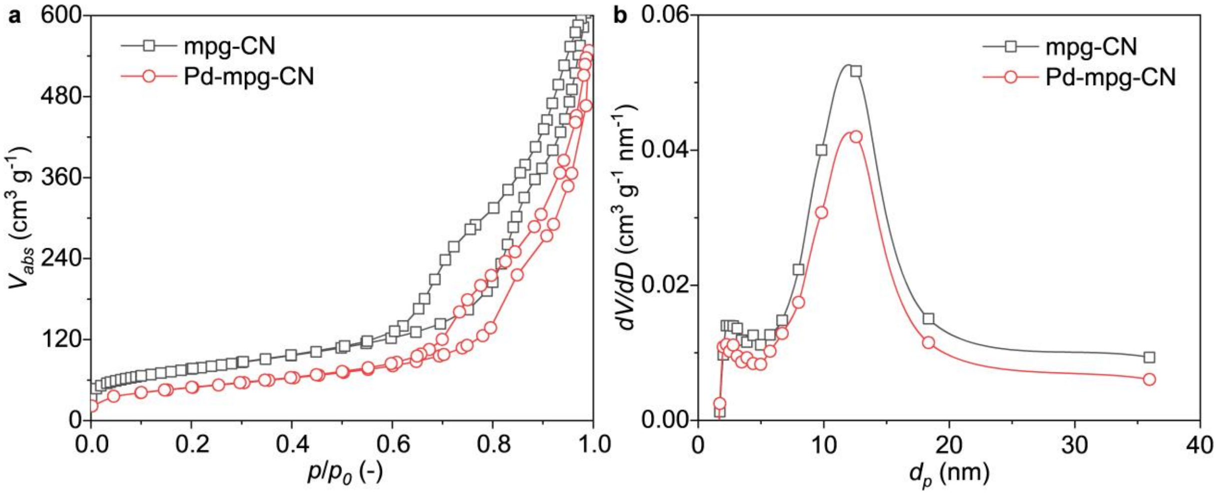

Figure S2. (a) Nitrogen adsorption-desorption isotherms and (d) the corresponding pore size distribution of mpg-CN and Pd-mpg-CN.

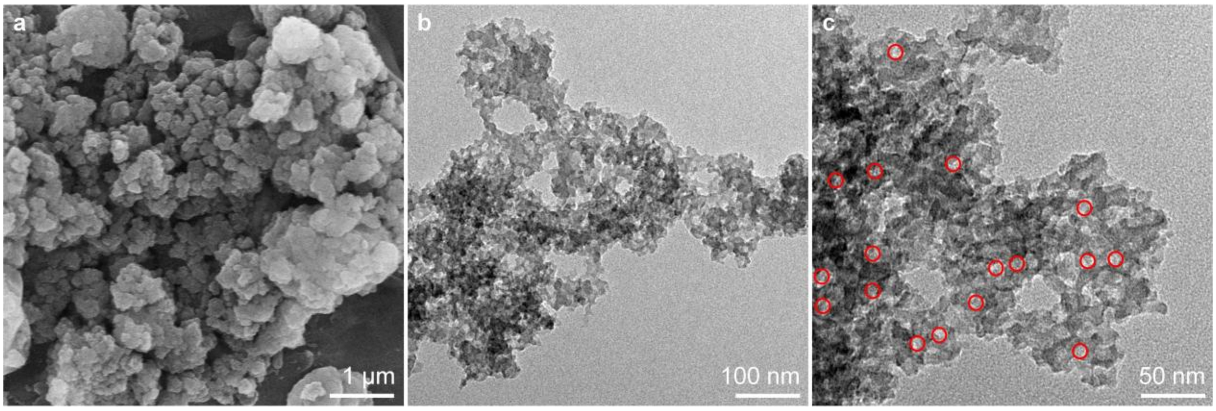

Figure S3. (a) SEM, (b) TEM, and (c) HRTEM images of mpg-CN. Red circles in (c) highlight the presence of mesopores.

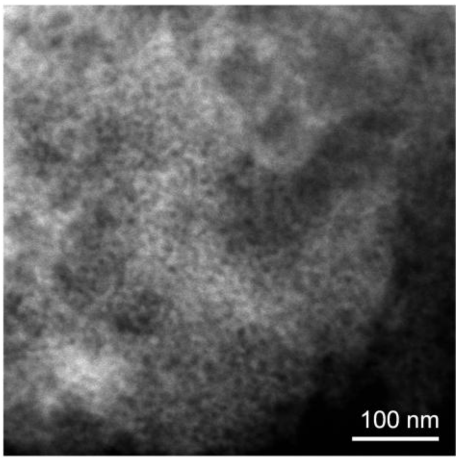

Figure S4. HAADF-STEM image of mpg-CN.

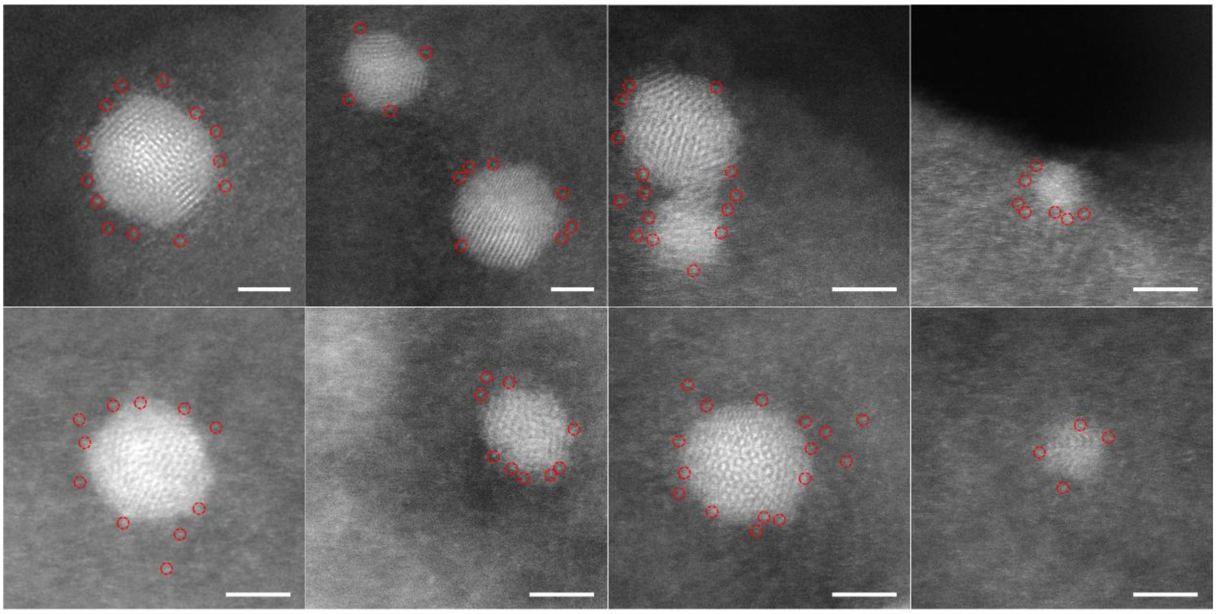

Figure S5. Additional AC-HAADF-STEM images of Pd-mpg-CN. All scale bars correspond to 2 nm. Isolated Pd atoms are highlighted by red circles.

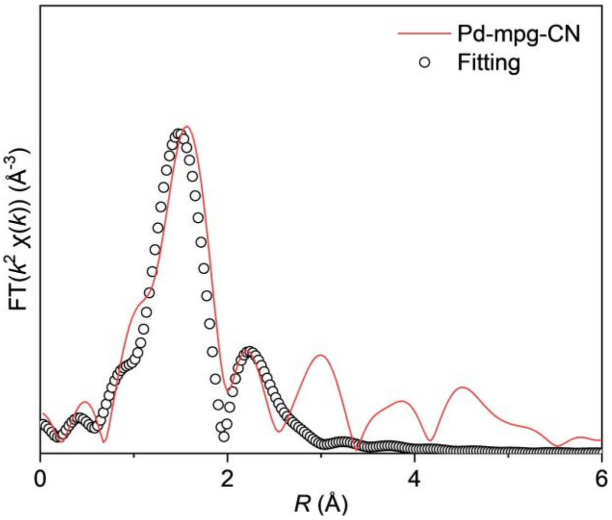

Figure S6. EXAFS fitting analysis for Pd-mpg-CN. Comparison between experimental and theoretical FT-EXAFS signal at the Pd K-edge.

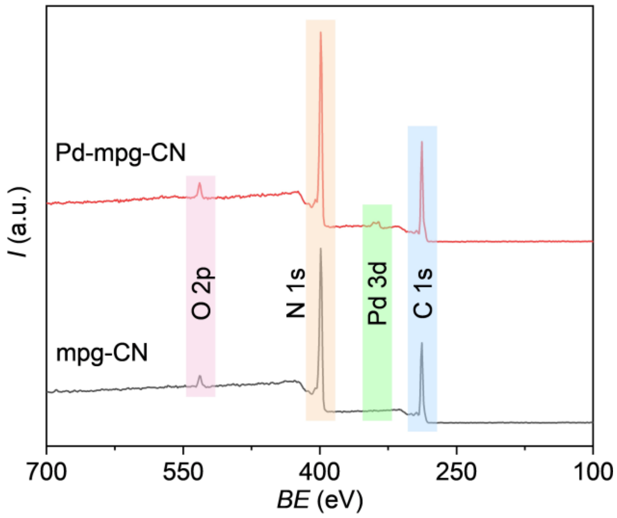

Figure S7. The XPS survey spectra and high-resolution of mpg-CN and Pd-mpg-CN.

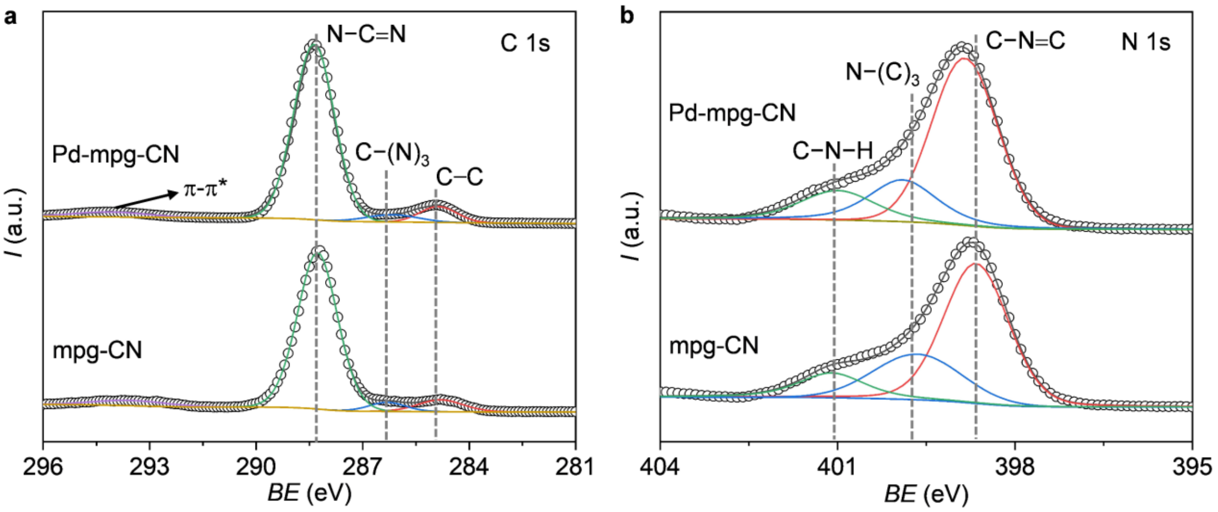

Figure S8. (a) C 1*s* and (b) N 1*s* XPS spectra of mpg-CN and Pd-mpg-CN.

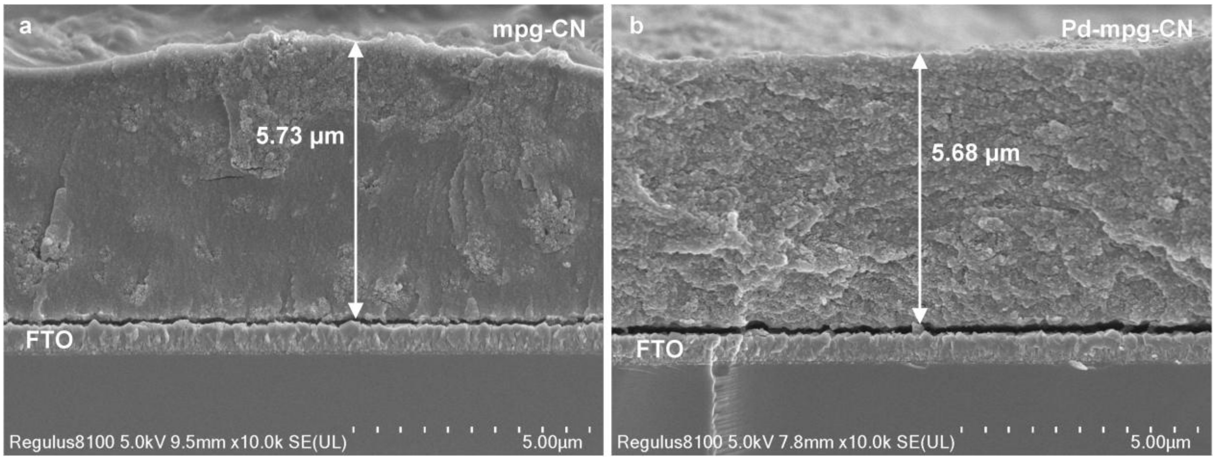

Figure S9. Cross-section SEM image of the FTO electrodes for mpg-CN and Pd-mpg-CN.

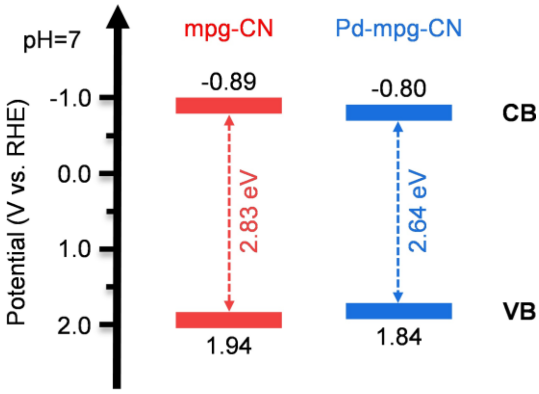

Figure S10. Electronic band structures of mpg-CN and Pd-mpg-CN.

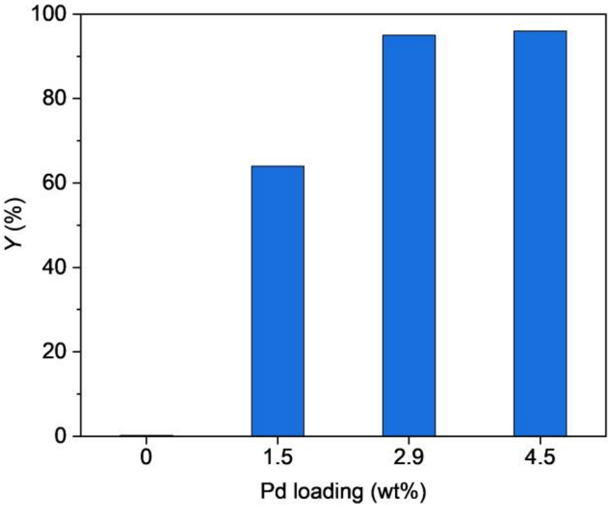

Figure S11. Effect of Pd loading in the hydrogenation of chalcone. Reaction conditions: catalyst (10 mg), chalcone (0.1 mmol), ultrapure water (2 ml), 1,4-dioxane (3 ml), triethylamine (TEA; 0.4 ml), blue light 40 W (λ=427 nm), reaction time (4 h), reaction temperature (313 K), reaction pressure (1 bar), N_2 atmosphere.

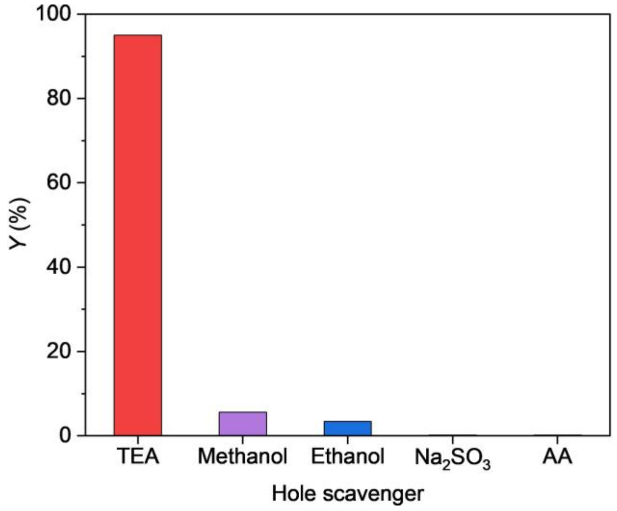

Figure S12. Comparison of the yields of 1,3-diphenylpropan-1-one production obtained in the hydrogenation of chalcone over Pd-mpg-CN using other representative hole scavengers: triethanolamine (TEA), methanol, ethanol, Na_2SO_3, and ascorbic acid (AA).

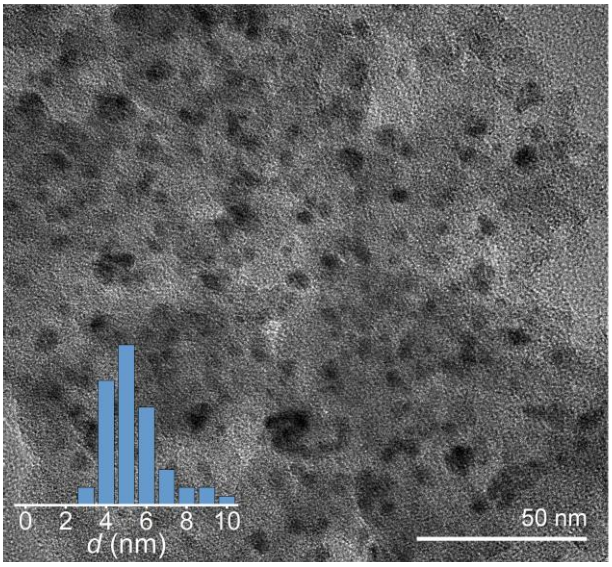

Figure S13. HRTEM image of the used Pd-mpg-CN after five consecutive reaction runs.

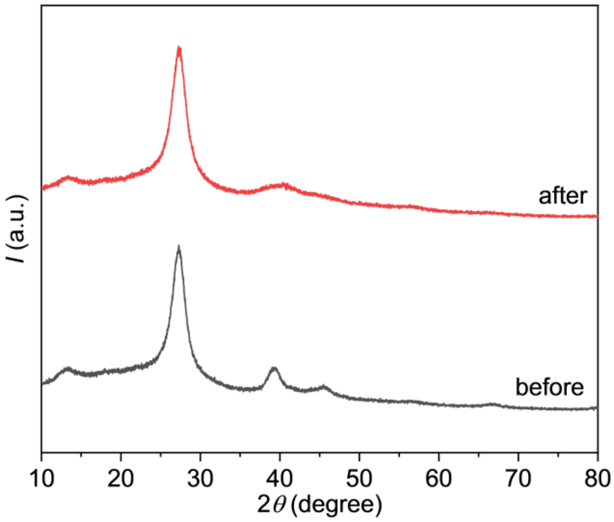

Figure S14. XRD patterns of Pd-mpg-CN before and after five consecutive reaction runs.

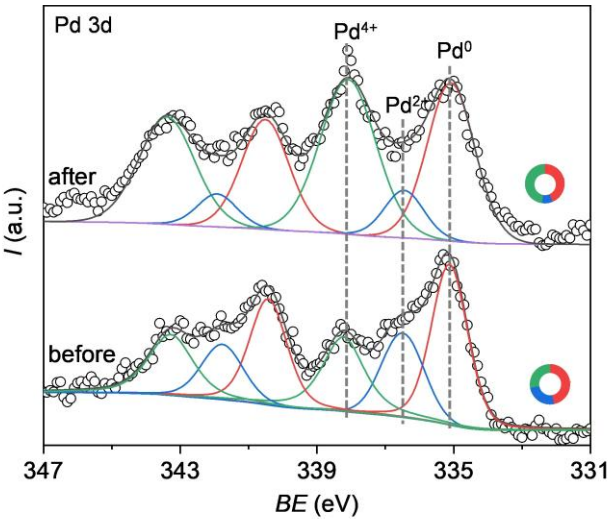

Figure S15. Pd 3*d* XPS spectra of Pd-mpg-CN before and after five consecutive reaction runs.

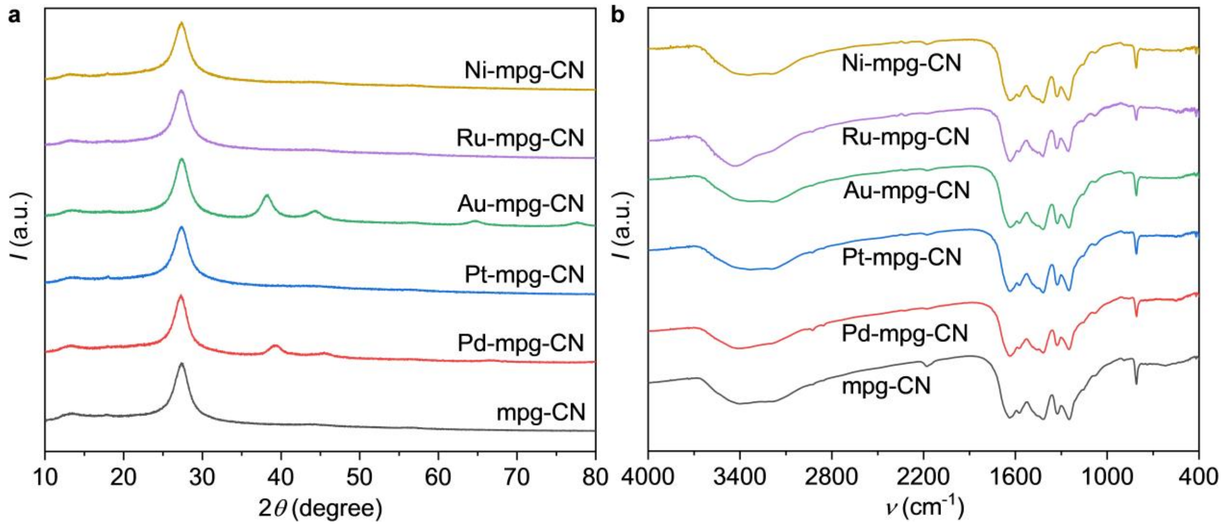

Figure S16. (a) XRD patterns and (b) FT-IR spectra of the different mpg-CN-supported catalysts.

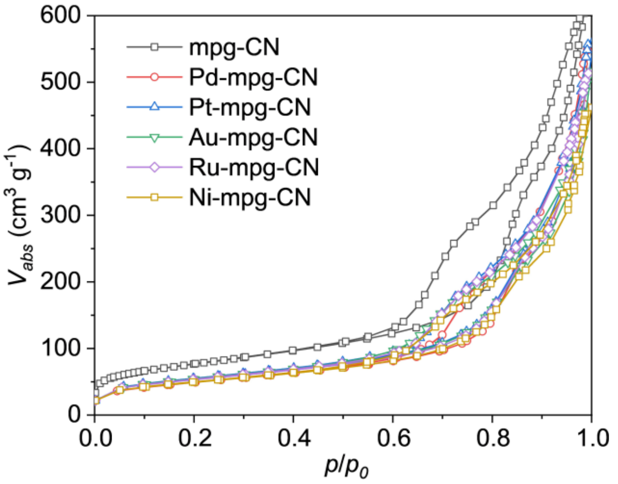

Figure S17. Nitrogen adsorption-desorption isotherms.

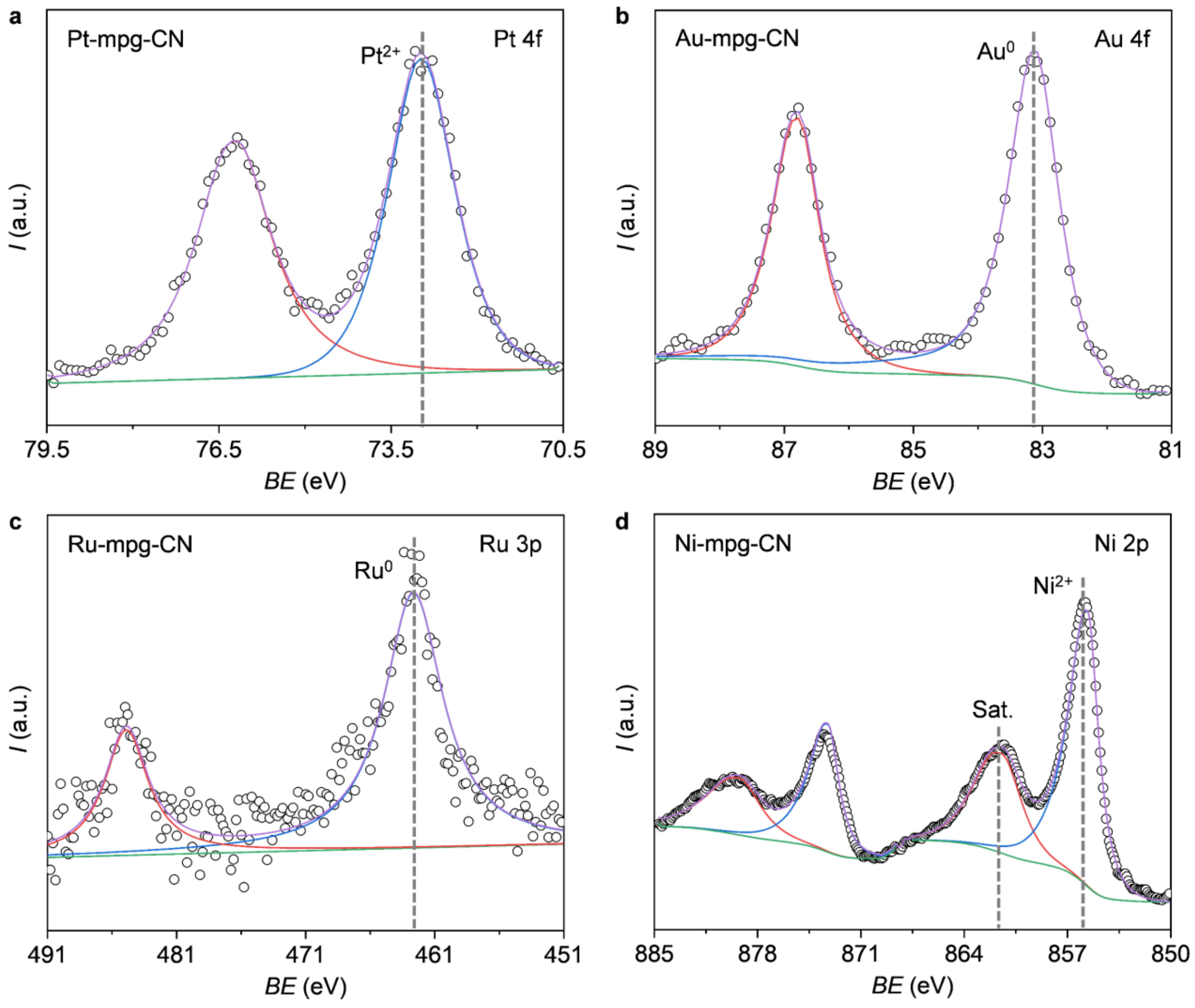

Figure S18. XPS spectra of (a) Pt-mpg-CN, (b) Au-mpg-CN, (c) Ru-mpg-CN, and (d) Ni-mpg-CN.

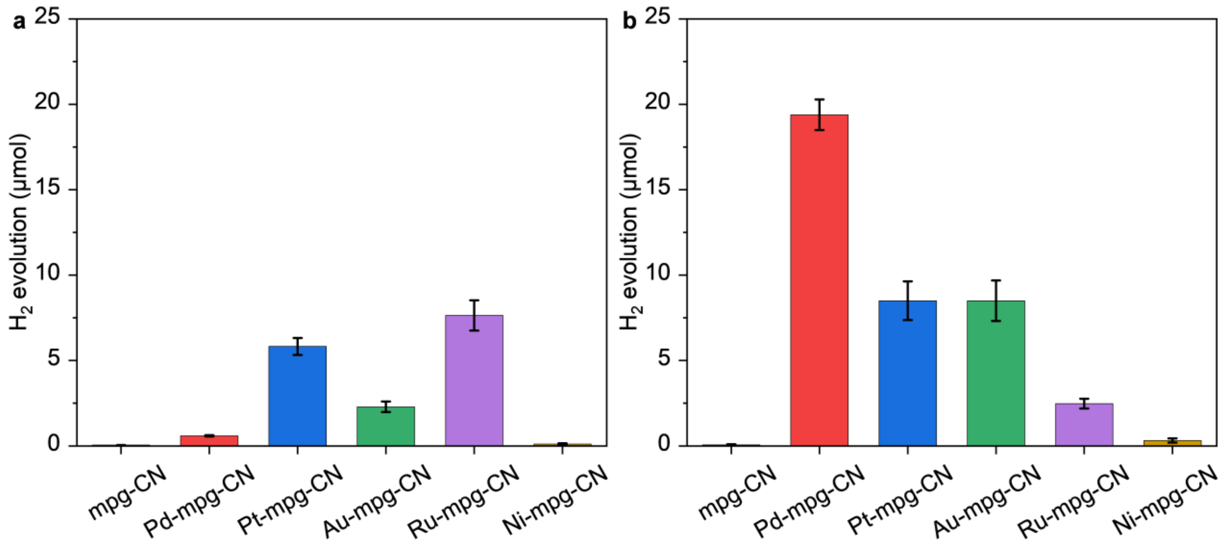

Figure S19. The amount of hydrogen evolved (a) with or (b) without organic substrate (chalcone).

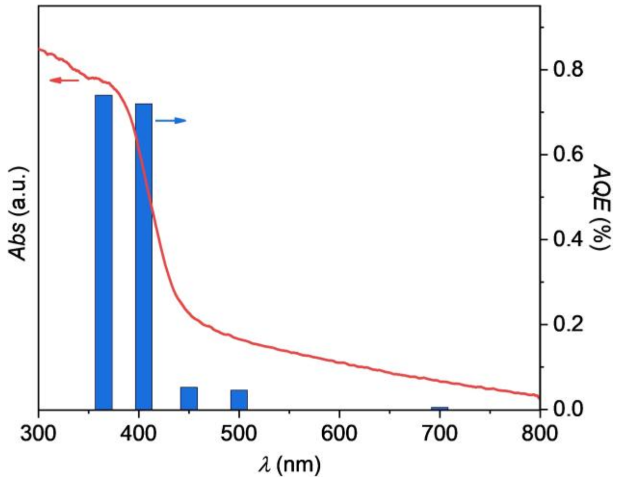

Figure S20. Wavelength-dependent AQEs of Pd-mpg-CN for photocatalytic water-donating selective transfer hydrogenation of chalcone. Reaction conditions: catalyst (10 mg), chalcone (0.1 mmol), ultrapure water (2 ml), 1,4-dioxane (3 ml), TEA (0.4 ml), 300 W xenon lamp equipped with different wavelength filters, reaction time (4 h), reaction temperature (313 K), reaction pressure (1 bar), N_2 atmosphere.

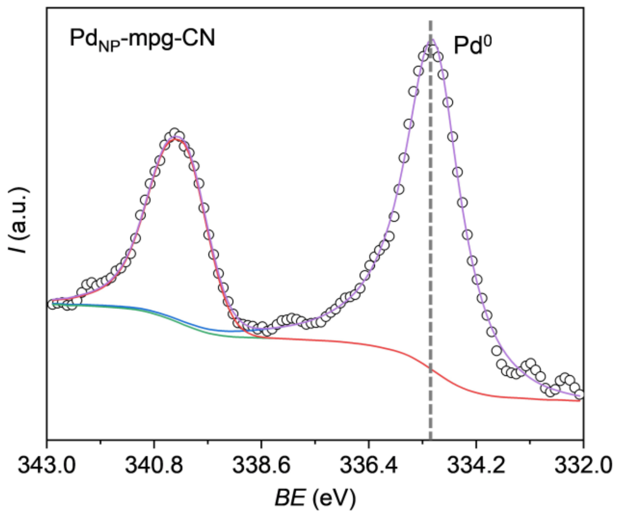

Figure S21. Pd 3*d* XPS of Pd$_{NP}$-mpg-CN.

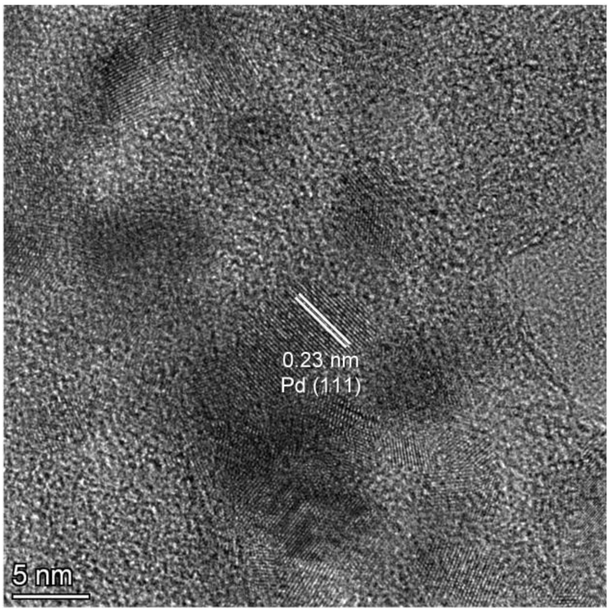

Figure S22. HRTEM images of Pd$_{NP}$-mpg-CN.

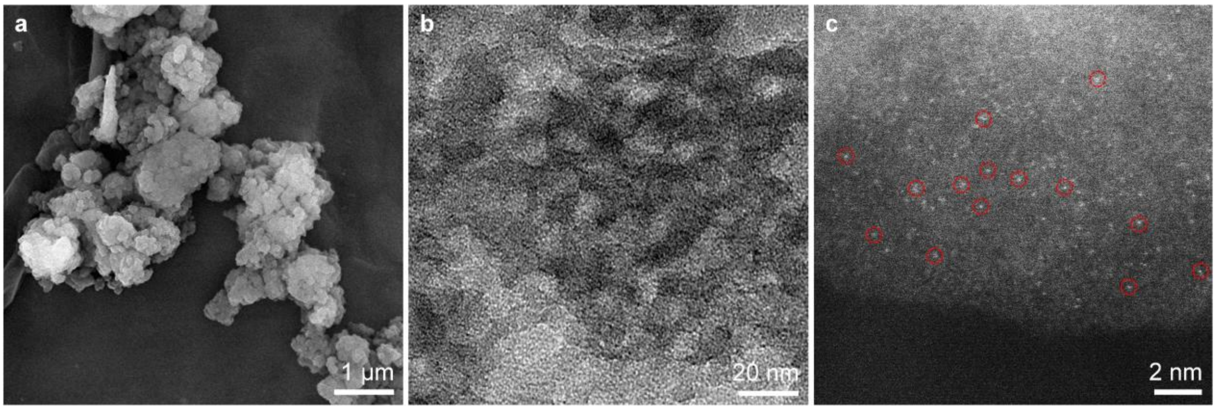

Figure S23. (a) SEM, (b) HRTEM, and (c) AC-HAADF-STEM images of Pd_1-mpg-CN.

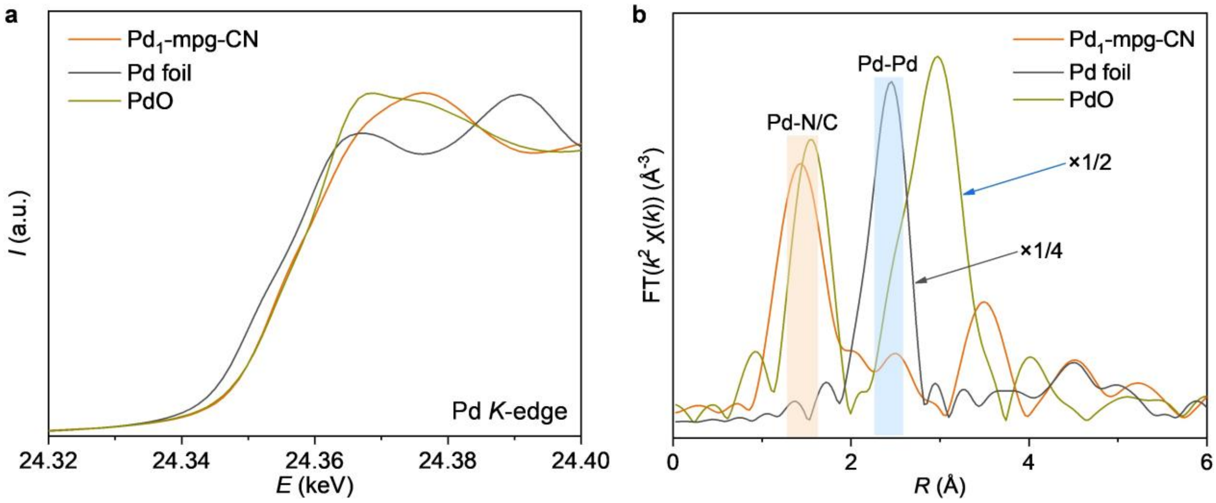

Figure S24. (a) Pd *K*-edge XANES spectra (b) Fourier transform of k^2-weighted Pd *K*-edge EXAFS spectra of Pd_1-mpg-CN. Pd foil and PdO were applied for comparison.

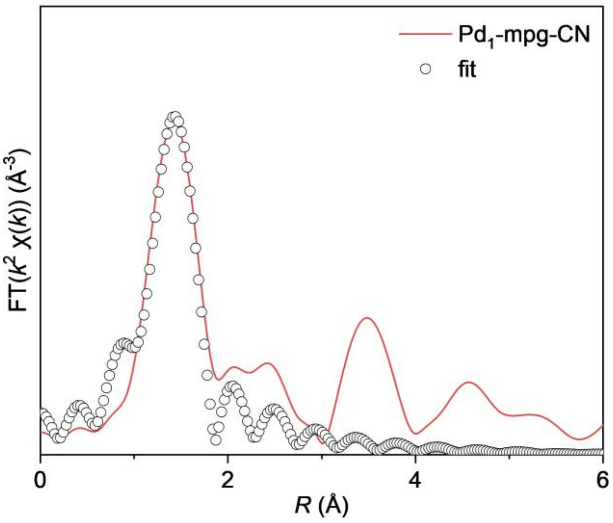

Figure S25. EXAFS fitting analysis for Pd_1-mpg-CN. Comparison between experimental and theoretical FT-EXAFS signal at the Pd K-edge.

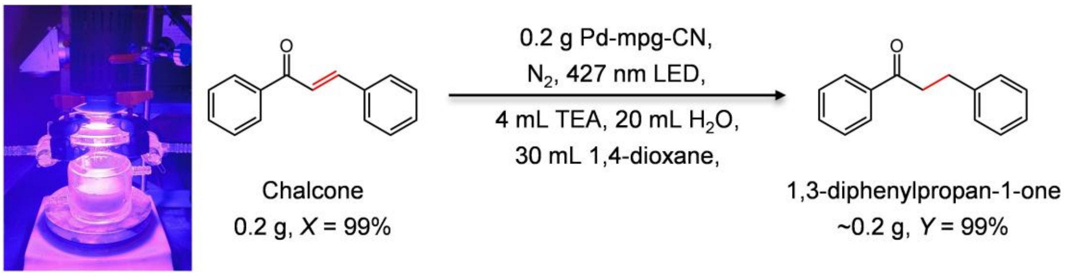

Figure S26. The scale-up experiment of Pd-mpg-CN on a gram scale.

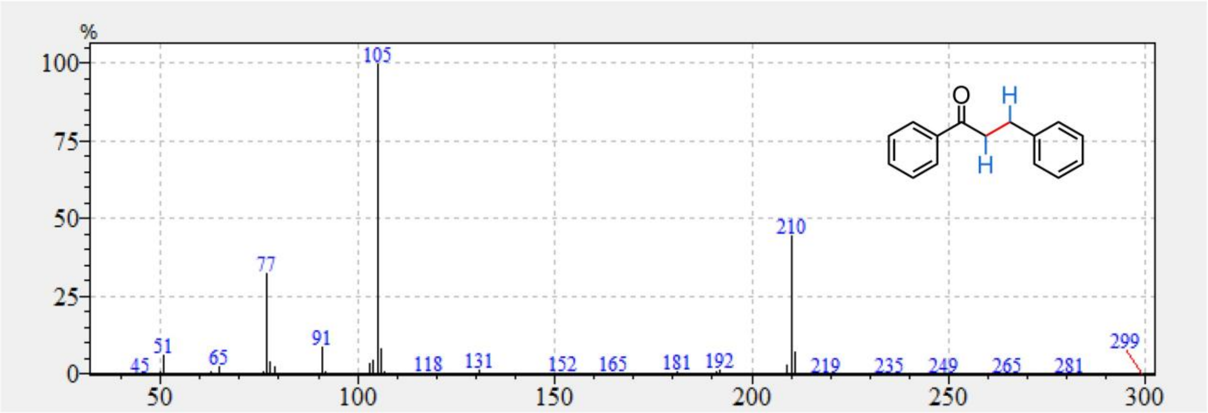

Figure S27. Mass spectrum of 1,3-diphenylpropan-1-one.

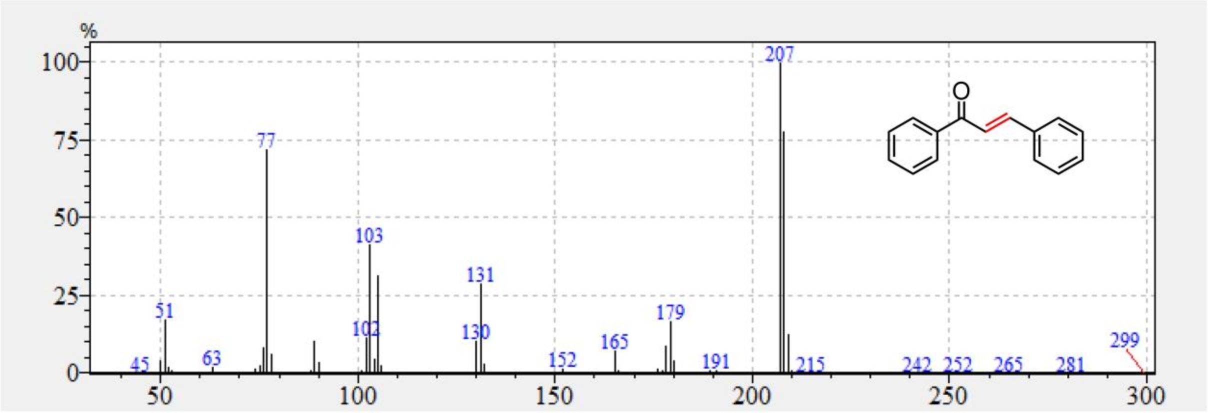

Figure S28. Mass spectrum of chalcone.

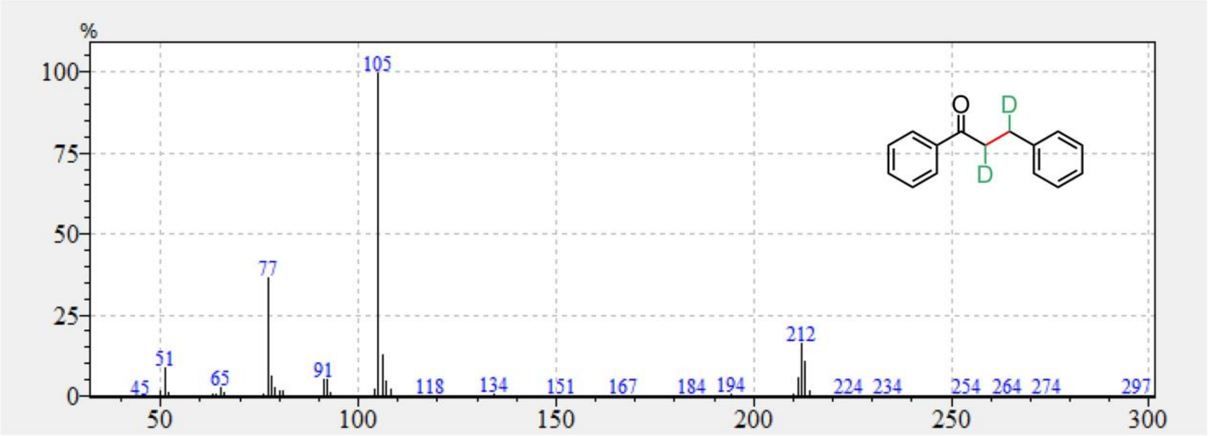

Figure S29. Mass spectrum of deuterated 1,3-diphenylpropan-1-one.

X-band EPR and light-induced EPR (LEPR) analysis of the Pd-mpg-CN catalytic system

1. X-band EPR spectra of neat mpg-CN and Pd-mpg-CN catalyst recorded in water with and without UV-light irradiation.

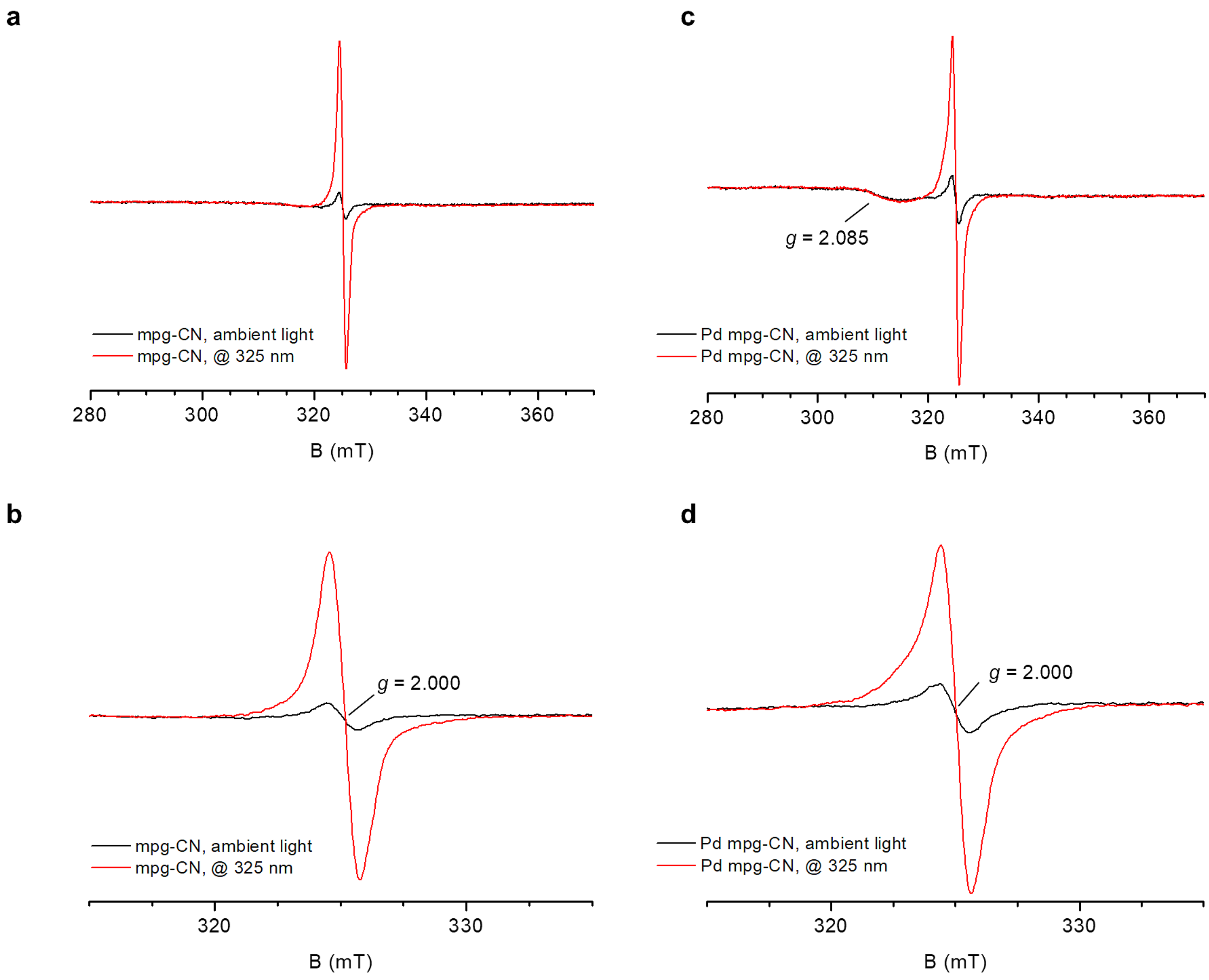

Figure S30. Continuous wave (CW) X-band EPR and light-induced EPR (LEPR) spectra of mpg-CN (**a**,**b**) and Pd-mpg-CN (**c**,**d**) in water recorded under ambient light (black spectra) and under 325 nm light irradiation (red spectra). Experimental conditions: ~9.080 GHz, 1.00 mW applied microwave power, 1.0 mT modulation width, 4 min scan time, 0.03 s time constant, T = 85 K. For the *in situ* LEPR experiments the excitation wavelength used was 325 nm (200 mW power source) and 5 min of light irradiation of the sample were used before recording the spectra.

The EPR spectrum of the Pd-mpg-CN in water recorded at ambient light conditions at T = 85 K is shown in Figure S30**c**,**d** (black line). Differing from the mpg-CN material (Figure S30**a**,**b**; black spectra) recorded in the same conditions, the resonance feature of the Pd-mpg-CN exhibits slightly broader wings

around the g = 2.000 resonance signal. The presence of closed-shell bound Pd cations (Pd (0) = [Kr] $4d^{10}$; Pd (II) = [Kr] $4d^8$, Pd (IV) = [Kr] $4d^6$) included in the mpg-CN framework, as observed in the XPS analysis, should not provide any EPR signal. However, in the low magnetic field region (around 310 mT), as shown in Figure S30**c** for Pd mpg-CN, a weak resonance appears at g = 2.085 (black spectrum, derivative-like signal). While most of the excess spins (e^-) arising from defect structure are located on C centres in both mpg-CN and Pd mpg-CN, spin polarization of the carbon framework and anisotropic distribution of the spin containing defects is significantly influenced by the presence of Pd species. The broader wing observed at g = 2.000 (Figure S30**d**, black spectrum) in Pd mpg-CN compared to neat mpg-CN (Figure S30**b**, black spectrum) arise from stronger perturbation of spin-polarized bonds that are in contact with Pd^0 sites (d^{10}). The weak signal at low field (around g = 2.085, Figure S30**c**, black spectrum) arises from the spin containing defects in the C framework that becomes perturbed by Pd cations single-atom sites and spin transfer may occur at these sites (leading to formation of small fraction of Pd^{3+}), even under ambient light illumination. Upon *in situ* irradiation at 325 nm, the observed EPR signal at g = 2.000 strongly increases in both mpg-CN and Pd mpg-CN (Figure S30**a-d**, red spectra); no clear effect, in terms of increasing of the EPR signal intensities, were detected in the low magnetic field region (Figure S30**a-d**, red spectra, below 310 mT). We can conclude that, upon light irradiation, the generated excess e^-, in the case of Pd mpg-CN, interacts more effectively with the Pd^0 nanoparticles than with single atom Pt sites. The photoexcited h^+ remains mostly delocalized over the entire mpg-CN framework, resulting in no clear changes of the EPR signal intensity (e.g. due to effective photo-oxidation of increasing numbers of Pt^{2+} sites to Pt^{3+} and/or even to Pt^{4+}), as seen from the unchanged EPR features expressed in the low magnetic field region.

2. X-band EPR spectra of the Pd-mpg-CN catalyst in water/1,4-dioxane mixture with and without UV-light irradiation.

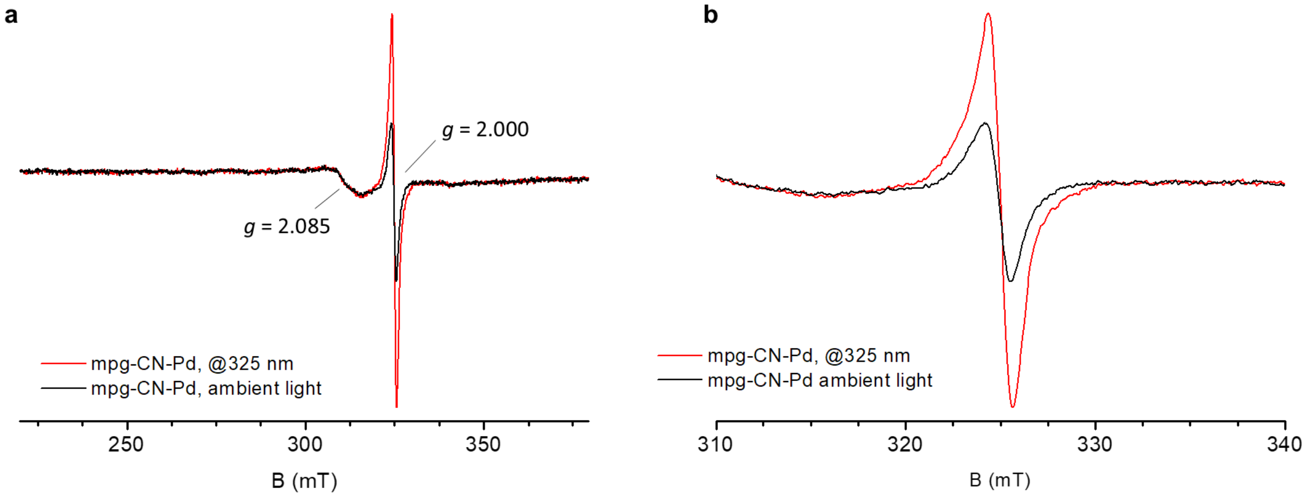

Figure S31. Continuous wave (CW) X-band EPR and light-induced EPR (LEPR) spectra (**a**,**b**) of Pd-mpg-CN in water/1,4-dioxane mixture recorded under ambient light (black spectra) and under 325 nm light irradiation (red spectra). Experimental: 9.081 GHz, 1.00 mW applied microwave power, 1.0 mT modulation width, 4 min scan time, 0.03 s time constant, $T = 85$ K. For the *in situ* LEPR experiment the excitation wavelength used was 325 nm (200 mW power source). The sample was subjected to 5 min of light irradiation before recording the LEPR spectrum.

The EPR spectrum of the Pd-mpg-CN in water/1,4-dioxane mixture recorded at ambient light conditions at $T = 85$ K is shown in Figure S31**a**,**b** (black spectrum). It displays nearly identical features as those observed in Pd-mpg-CN in water, given in Figure S30, with anisotropic resonance signal around *g* value of 2.000, ΔB_{pp} of 1.3 mT, and better pronounced derivative signal at $g = 2.085$. Similarly, it is observed an increase in the EPR signal intensity upon *in situ* @325 nm irradiation in the magnetic field region of the excited e^- ($g \sim 2.000$), with no clear variation of the EPR signal in the low-field region, where interaction of h^+ with Pd single atom sites are expected to occur (Figure S31**a**,**b**; red spectra). From these results, the addition of 1,4-dioxane to the water solution does not substantially alter the electronic distribution of the photogenerated e^-/h^+ spins compared to the spectra seen in the Pd-mpg-CN when the EPR signals were recorded in neat water.

3. In operando LEPR spectra of the Pd-mpg-CN catalyst in water and water/1,4-dioxane mixture using an EPR signal acquisition sequence of “light off – UV light on – light off”.

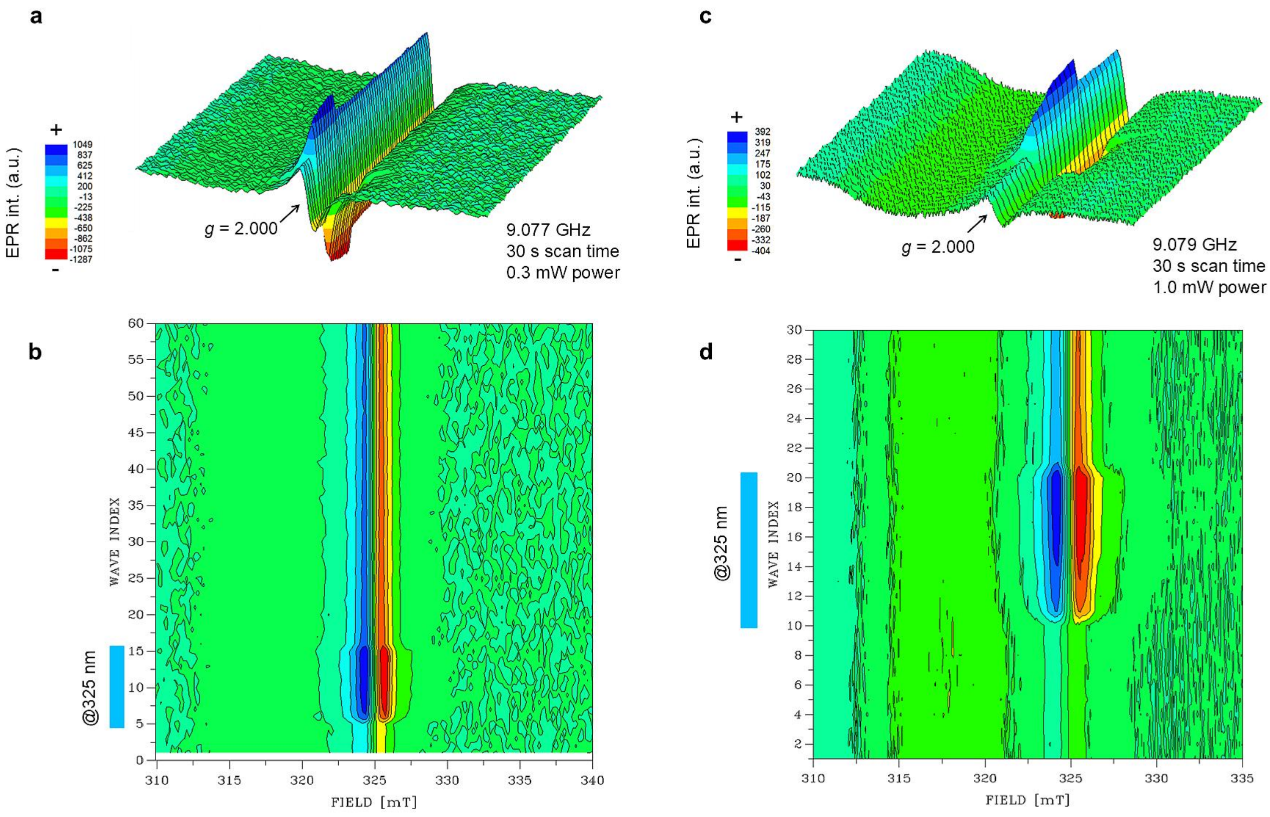

Figure S32. *In operando* 3D (**a**,**c**)/2D (**b**,**d**) LEPR spectra of Pd-mpg-CN dispersed in water (**a**,**b**) and in water/1,4-dioxane mixture (**c**,**d**). The spectra show that as soon as UV light is applied *in situ* to the sample kept in the cavity resonator (T = 85K) a strong resonance signal emerges in the g = 2.000 region, which corresponds to effective formation of photoexcited e^-. Upon cutting off the UV light, part of the photoexcited electrons recombine fast with the holes, leaving a residual resonance signal that corresponds to formation of stable (at T = 85 K) -C$^•$…H$^+$ intermediate (with C that belongs to the mpg-CN framework). Each wave index shown in panels **b** (Y-axis, wave 1-60) and **d** (Y-axis, wave 1-30) corresponds to the sequential spectrum acquired by using 30s scan time and 0.03 s time constant.

4. X-band EPR spectra of the Pd-mpg-CN catalyst in water/1,4-dioxane/TEA mixture with and without UV-light irradiation.

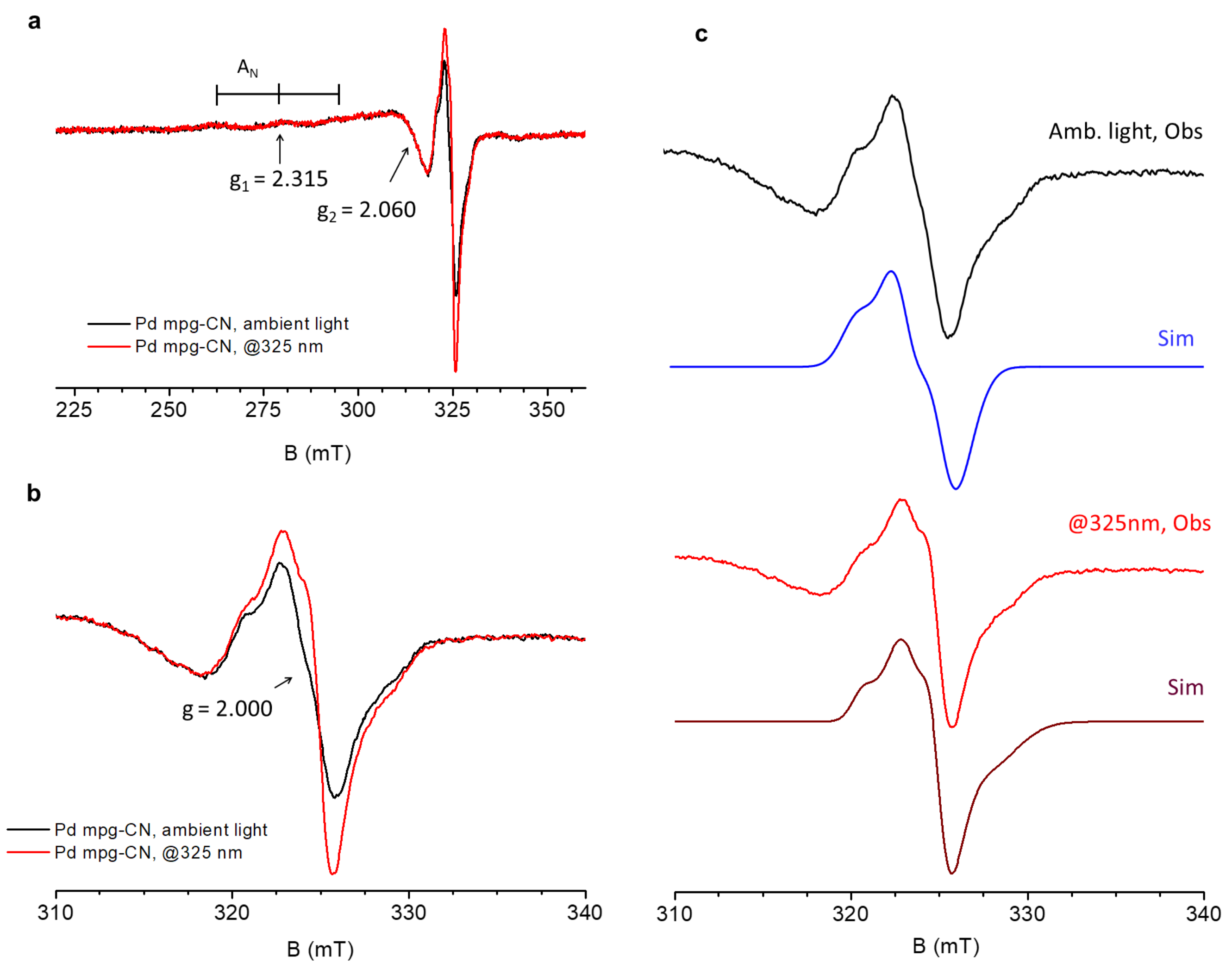

Figure S33. Continuous wave (CW) X-band EPR and light-induced EPR (LEPR) spectra (**a**,**b**) of Pd-mpg-CN in water/1,4-dioxane/TEA mixture recorded under ambient light conditions (black spectra) and under 325 nm light irradiation (red spectra). Experimental: 9.077 GHz, 1.00 mW applied microwave power, 1.0 mT modulation width, 4 min scan time, 0.03 s time constant, $T = 85$ K. For the *in situ* LEPR experiment the excitation wavelength used was 325 nm (200 mW power source). The sample was subjected to 5 min of light irradiation before recording the LEPR spectrum. Panel **c** gives the computer simulation (blue spectrum and wine spectrum) of the anisotropic EPR resonance signal (ambient light and @325nm) of the radical specie in the $g = 2$ region, according to the spin-Hamiltonian parameters given in text.

The EPR spectrum of the Pd-mpg-CN in water/1,4-dioxane/TEA mixture recorded in ambient light conditions at 85 K is shown in Figure S33**a**,**b** (black spectrum). A highly anisotropic signal around g =

2.000 and a strong axial signal developing at $g > g_e = 2.0023$ are observed. The signal at $g > g_e$ accounts for the clear coupling between the $S = 1/2$ spin moment and one ^{14}N nuclei from TEA, affording a hyperfine (A_N) term of 17.8 mT and system axial anisotropy of $g_1 = 2.315$ and $g_2 = 2.060$. This property is consistent with localization of the $S=1/2$ onto a Pd^{3+} centre (P. Stathi, L. Belles, Y. Deligiannaki. Multipotent Atomic Palladium Species Pd^{1+}, $Pd^{2+}–O^{2-}$, and Pd^{3+} Formed at the Interface of Pd/TiO_2 Nanoparticles: Electron Paramagnetic Resonance Study. *J. Phys. Chem. C* **2022**, *126*, 14125-14137). The signal around $g = 2.000$, already at ambient light, became highly anisotropic and contains a resolved ^1H nuclear hyperfine arising from $H^•$ generated from dissociation of water molecules. The above results indicate that TEA enhances efficient spin polarization of the bonds around the spins containing C centres of the mpg-CN support and may facilitate the dissociation of water at the Pd single-atom sites (into OH^- and H^+) with further binding/interaction of H^+ to Pd nanoparticles. These H^+ become active to accept the photoexcited e^-. Moreover, we propose that the stronger spin polarization induced by TEA on the mpg-CN framework contributes to the enhancement of the localization of h^+ near the $Pd^{2+/4+}$ single atoms and to the resulting fast oxidation of Pd^2 to Pd^{3+}. Upon *in situ* irradiation in the EPR cavity resonator at 325 nm, the EPR signal only slightly increased in the $g \sim 2.000$ region (Figure S33**a**-**b**, red spectrum), showing changes in the signal's shape compared to that one recorded at ambient light. There was no indication of any substantial changes in the EPR signal in the $g > g_e$ region. Simulation of the resonance envelope in the $g \sim 2.000$ region (Pd^0….$H^•$) at ambient light and under irradiation are given in Figure S33**c** (blue and wine spectra), which provide the estimated g-tensor and A-tensor components for the Pd^0….$H^•$ specie without and under 325 nm irradiation. At ambient light the EPR simulation gives the following values: $g_x = 2.018$, $g_y = 2.002$, $g_z = 1.993$; ^1H: $A_x = 2.30$ mT, $A_y = 2.10$ mT, $A_z = 1.46$ mT. Lorentzian/Gaussian ratio = 1.0, line-width tensor of $L_x = 1.7$ mT, $L_y = 1.5$ mT and $L_z = 2.0$ mT. Under 325 nm light irradiation the resonance line, besides the spin-Hamiltonian values reported above, contains the additional contribution (~9% of the total spin) of excess e^- (centred on C from the mpg-CN framework) with g-tensor parameters of $g_\perp = 2.000$ and $g_{//} = 1.980$. This result indicates that the electrons (e^-) interacts with H^+ (acceptor) and then with the Pd nanoparticles, whereas the excess of h^+ become localized onto the single Pd atoms. Moreover, because the number of Pd^{2+} centre is less compared to the sum of Pd^0 and Pd^{4+}, according to XPS results, h^+ are consumed faster than the photogenerated e^-, which in turn should prevent the fast recombination of e^-/h^+ during light-irradiation, and ultimately favour catalysis, with formation of $H^•$ and Pd^{3+} spin active intermediates.

5. X-band EPR spectra of the Pd-mpg-CN catalyst in water/1,4-dioxane in the presence of chalcone without and with UV-light irradiation.

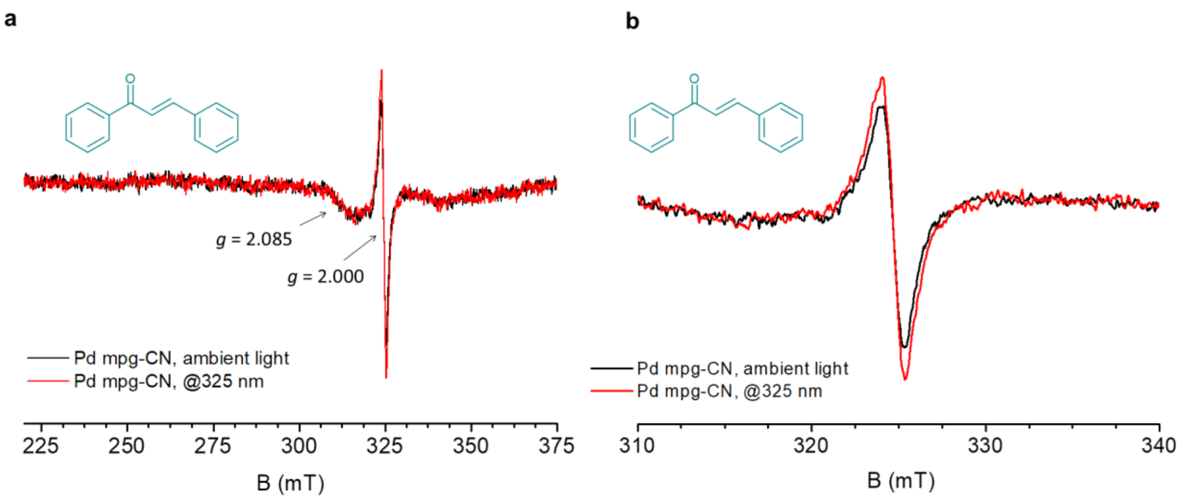

Figure S34. Continuous wave (CW) X-band EPR and light-induced EPR (LEPR) spectra (**a**,**b**) of Pd-mpg-CN in water/1,4-dioxane mixture in the presence of substrate (chalcone) recorded under ambient light (black spectra) and under 325 nm light irradiation (red spectra). Experimental: 9.075 GHz, 1.00 mW applied microwave power, 1.0 mT modulation width, 4 min scan time, 0.03 s time constant, $T = 85$ K. For the *in situ* LEPR experiment the excitation wavelength used was 325 nm (200 mW power source). The sample was subjected to 5 min of light irradiation before recording the LEPR spectrum.

The EPR spectrum of the Pd-mpg-CN in water/1,4-dioxane mixture in the presence of the substrate (chalcone) recorded at ambient light conditions at $T = 85$ K is shown in Figure S34 (**a**,**b**; black spectra). It shows a very weak anisotropic resonance feature at $g = 2.000$ with ΔB_{pp} of 1.3 mT, which is almost identical to that observed in Pd-mpg-CN recorded in water (Figure S30**a**,**b**; black spectra) or water/1,4-dioxane mixture (Figure S31, black spectrum) in the absence of substrate. From the observed resonance signals, the chalcone molecule does not alter electronic and spin configuration of the Pd nanoparticles. However, chalcone slightly perturb the Pd single-atom sites, possibly through the C=O group, which has similar O donor proclivity as those expressed by water or 1,4-dioxane molecules. This is consistent with the minor perturbation of the derivative resonance observed in the low-field region, as seen at $g = 2.085$. Upon *in situ* irradiation at 325 nm, only an extremely weak enhancement of the EPR signal was observed at $g = 2.000$ (Figure S34**a**,**b**; red spectra), without variation of the signal anisotropy. These results indicate that two scenarios can occur when the substrate is added to the system but without addition of TEA: **i)** the excited e^- and h^+ are fast formed under UV light irradiation, and fast transferred to produce closed shell hydride

anions (H^-) and closed-shell Pd single atom species, and/or **ii)** the photoexcited electrons that drive the generation of hydride anions, and the hydrogenation of the substrate, are very inefficient, because of their fast recombination. Based on the catalytic results that showed the key role for the presence of TEA molecules to drive catalysis in the hydrogenation process, the scenario (**ii**) is validated. Moreover, the result suggests that TEA could function either as an additional hydride source during catalysis, besides water, can solely acts as electronic coupling mediator that gate easier Pd^{2+}/Pd^{4+} red-ox switch, or function through combination of all these effects. Further insides about the TEA role in catalysis is unveiled in the next paragraphs.

6. X-band EPR spectra of the Pd-mpg-CN catalyst in water/1,4-dioxane/TEA in the presence of chalcone substrate recorded with and without UV-light irradiation.

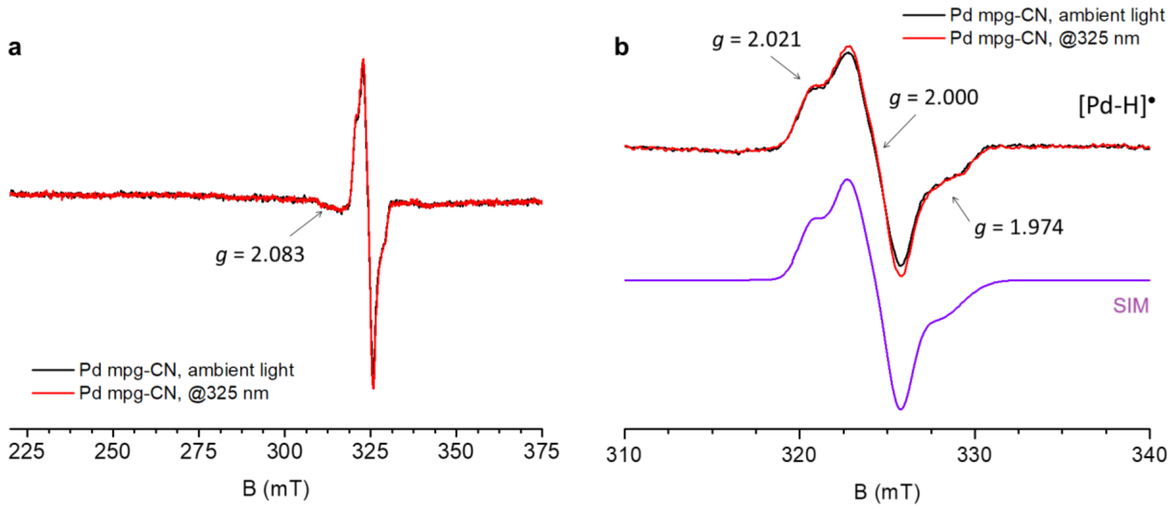

Figure S35. Continuous wave (CW) X-band EPR and light-induced EPR (LEPR) spectra of Pd-mpg-CN in water/1,4-dioxane/TEA mixture (**a**,**b**) in the presence of substrate (chalcone) recorded without (black spectra) and with (red spectra) light irradiation. Experimental: 9.073 GHz, 1.00 mW applied microwave power, 1.0 mT modulation width, 4 min scan time, 0.03 s time constant, $T = 85$ K. For the *in situ* LEPR experiment the excitation wavelength used was 325 nm (200 mW power source). The sample was subjected to 5 min of light irradiation before recording the LEPR spectrum. The violet spectrum in panel **b** corresponds to the simulated EPR signal under light irradiation, being almost identical to that recorded under ambient light.

When all the reactants were present in the reaction mixture (water/1,4-dioxane/TEA/chalcone), the EPR spectrum of the Pd-mpg-CN recorded under ambient light conditions at $T = 85$ K expressed characteristic features (Figure S35**a**,**b**; black spectra). The resonance signal around $g = 2.000$ shows a resolved anisotropic g-tensor, giving values from simulation of the resonance envelope (Figure S35**b**, violet spectrum) at $g_x = 1.974$, $g_y = 2.000$ and $g_z = 2.021$ (line-width tensor $L_x = 3.6$ mT, $L_y = 1.0$ mT, $L_z = 2.7$ mT, Lorentzian/Gaussian ratio = 1.0). Notably, the absence of ^{1}H hyperfine signals is here observed (compare with Figure S33, black lines), suggesting that the Pd-H$^•$ system ($S = ½$) has dominant metal character, strong interaction with Pd centre and the magnetic coupling between the electron spin moment with the ^{1}H nuclei is obscured by strong dipolar broadening. The other EPR signal observed at magnetic field > g_e (< 320 mT) is extremely weak, which is consistent with the presence of diamagnetic $Pd^{2+/4+}$ single-atom sites. Upon irradiation at 325 nm, almost no changes in any region of the EPR spectrum can be detected (Figure S35**a**,**b**;

red spectrum). Such feature indicates that reaction between h^+ and the Pd single atom centres is very fast, and no Pd^{3+} intermediate oxidation state can be observed when all the reactants are present in the environment. On the other hand, the formation of $[Pd\text{-}H]^{\bullet}$ (EPR active specie, $S = 1/2$) approaches a steady state condition, as seen from the witnessed near invariance between ambient light and under light irradiation (@325 nm); its further reduction of the $[Pd\text{-}H]^{\bullet}$ by photoexcited e^-, leading to the formation of the hydride form $[Pd\text{-}H]^-$ (EPR silent specie, $S = 0$), destabilizes the $Pd\text{-}H^-$ interaction, allowing hydride transfer to the substrate, which is considered the slow kinetic processes.

7. X-band EPR spectra of the Pd-mpg-CN catalyst in D_2O/1,4-dioxane/TEA in the presence of chalcone substrate recorded with and without UV-light irradiation

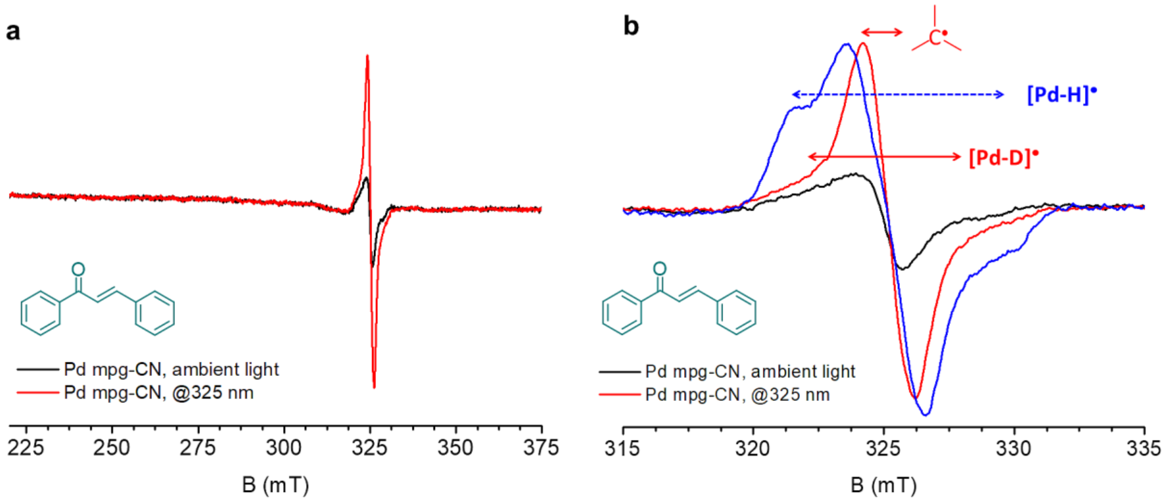

Figure S36. Continuous wave (CW) X-band EPR and light-induced EPR (LEPR) spectra (**a**,**b**) of Pd-mpg-CN in D_2O/1,4-dioxane/TEA mixture in the presence of substrate (chalcone). Experimental: 9.071 GHz, 1.00 mW applied microwave power, 1.0 mT modulation width, 4 min scan time, 0.03 s time constant, T = 85 K. For the *in situ* LEPR experiment the excitation wavelength used was 325 nm (200 mW power source). The sample was subjected to 5 min of light irradiation before recording the LEPR spectrum. The blue spectrum in panel **b** is the correspondent irradiated spectrum from Pd-mpg-CN in water/1,4-dioxane/TEA mixture (shown in Figure S35) that has been frequency corrected for easier comparison.

We further probe the effect on the EPR spectra brought by substitution of water with D_2O in the 1,4-dioxane/TEA/chalcone substrate. The EPR spectrum of the Pd-mpg-CN was recorded initially under ambient light conditions at T = 85 K and exhibited a clear anisotropy around g = 2.000 (Figure S36**a**,**b**; black spectrum). The recorded resonance contains the signal of $C^\bullet$-centre (isotropic) and the anisotropic signal of $[Pd\text{-}D]^\bullet$. Upon light irradiation (Figure S36**a**,**b**; red spectrum), there is a clear accumulation of photoexcited spin states centred on C-atoms (e^-, S = ½) compared to that witnessed in H_2O (resonance spectrum is added here for quick comparison, as blue spectrum). Moreover, the magnetic field spread decreases in $[Pd\text{-}D]^\bullet$ compared to $[Pd\text{-}H]^\bullet$.

The deuterium nucleus has a higher nuclear spin (I = 1) but 6.5-fold lower hyperfine coupling constant (HFC) than ^1H, thus the EPR signal spread must decrease upon using deuterated water, as witnessed here. Therefore, the proton source that drives the hydrogenation reaction originates from water, not from TEA. Furthermore, the reduction by photoexcited e^- of D^+ to $D^\bullet$ is clearly, by far less efficient, compared to H^+.

Nevertheless, there is no evidence of radical formation due to TEA decomposition/reaction with h^+/e^- species. Thus, TEA acts as a key molecule that facilitates e^- migration to Pd nanoparticles and/h^+ to Pd single atoms and appears to aid H^- spillover process from the Pd nanoparticles to the Pd single-atom sites where is bound the substrate molecule and where the oxidation reaction by h^+ of Pd^{2+} to Pd^{4+}occurs.

8. X-band EPR spectra of the Ni-mpg-CN catalyst in H_2O/1,4-dioxane and H_2O/1,4-dioxane/TEA in the presence of chalcone substrate, with and without light irradiation.

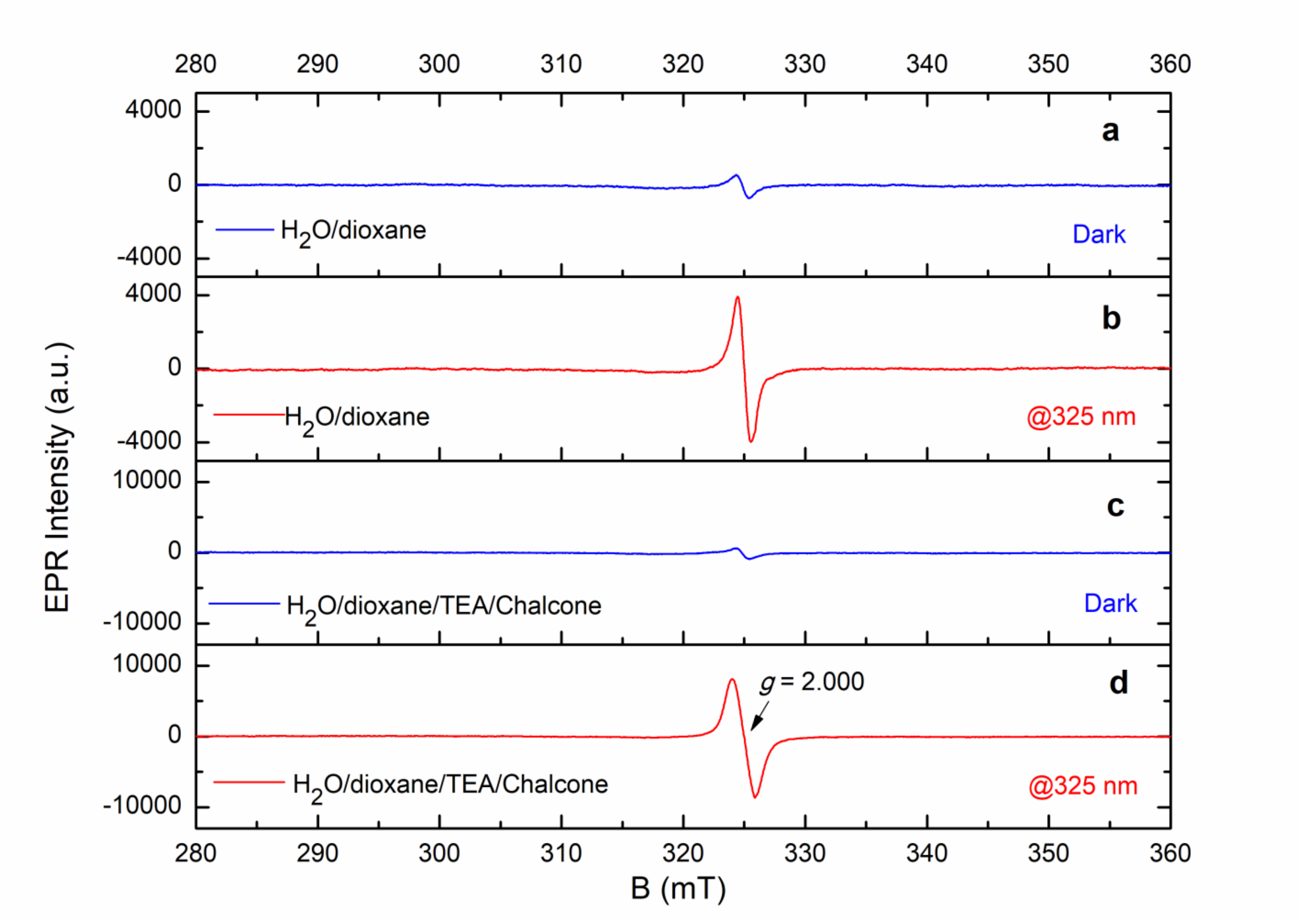

Figure S37. Continuous wave (CW) X-band EPR and light-induced EPR (LEPR) spectra of Ni-mpg-CN recorded in water/1,4-dioxane (**a**,dark; **b**, light-irradiation) and in water/1,4-dioxane/TEA mixture in the presence of substrate (chalcone) (**c**,dark; **d**, light-irradiation). Experimental: 9.078-9.079 GHz, 0.6 mW applied microwave power, 0.8 mT modulation width, 4 min scan time, 0.03 s time constant, $T = 90$ K. For the *in situ* LEPR experiment the excitation wavelength used was 325 nm (200 mW power source). The sample was subjected to 5 min of light irradiation before recording the LEPR spectrum.

Note: The Ni^{2+} usually adopts the low spin state and is EPR silent (S=0, $3d^8$), thus the EPR spectra observed in Ni-mpg-CN shown in Figure S37 are consistent with retention of such oxidation state, irrespective of the conditions, dark to light irradiation. High spin state in Ni^{2+}, a non-Kamer system (S=1), has large zero-field-splitting and it is also undetectable at X-band frequency.(1) The presence of paramagnetic states for Ni, such as Ni^{+} and Ni^{3}+ are excluded here, even when the system is monitored by EPR technique under turn-over (e.g. in presence of substrate and under light irradiation). For example, irradiation of the Ni^{2+}-EDTA

water suspensions (T = 77 K, ^{60}Co γ-source) gives paramagnetic Ni^{3+}-EDTA (S = 1/2) that exhibits EPR signatures at g_z = 2.336 and g_x = g_y = 2.139.(2) Ni^{3+} complexes obtained by in-situ oxidation of Ni^{2+} with magic blue and cerium ammonium nitrate can give EPR signatures for Ni^{3+} with large g-tensor values, as large as g(x,y,z) = 2.32, 2.23, 2.01.(3) The Ni^{3+} cation in NiSOD(4) exhibits EPR spectrum with S=1/2 signal at g_x=2.30, g_y=2.24, and g_z=2.01. In NiFe Hydrogenase, (5) the measured g-values for Ni^{3+} cations are 2.33, 2.16 for Ni-B and 2.32, 2.24 for Ni-A. Moreover, the presence of Ni^{+} paramagnetic cations in materials and organometallic complexes also exhibit very clear resonance signatures, at $g_{//}$ ~ 2.20-2.40 and $g_{\perp}$~2.06-2.10.(6) Therefore, in Ni-mpg-CN the oxidation (+2) state of Ni does not change in presence of the chalcone substrate and under light irradiation, in agreement with the catalytic inefficiency of the system to carry out transfer hydrogenation reactions.

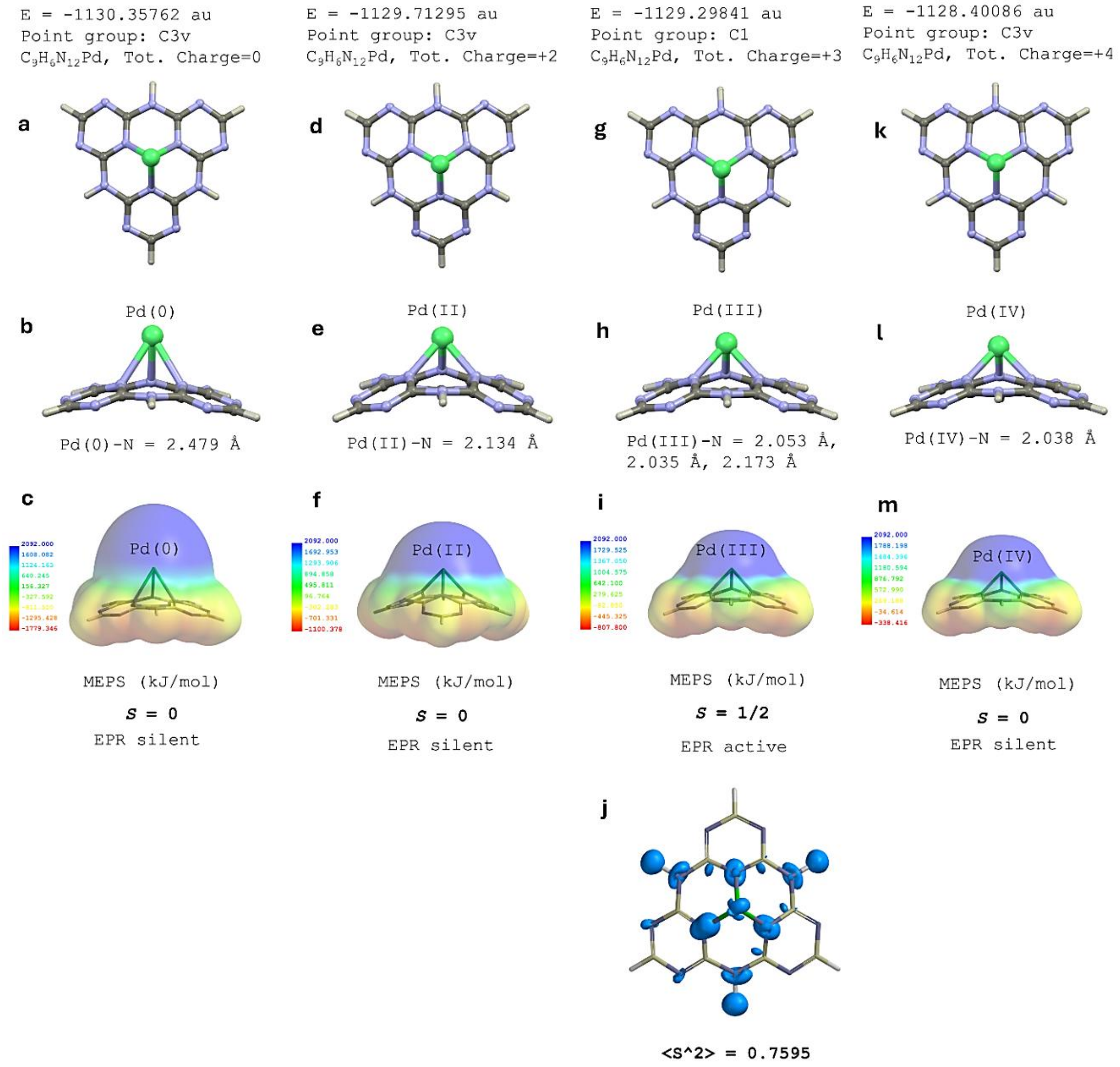

Figure S38. Theoretical calculations of the geometry optimized models showing the Pd species with distinct valence states (i.e., 0, 2+, 3+, and 4+) interacting with the triazine-based units of carbon nitride ($C_9N_{12}H_6Pd$, coded in the manuscript as Pd mpg-CN). Geometry optimization were performed by density functional theory (DFT) in the gas phase using B3LYP (panel **a,b,c,d,e,f,k,l,m**) or BP86 functional (panel **g,h,i,j**) with the Euler-Maclaurin-Lebedev grid (70, 302) and basis set 6-31G* for C,H,N and the effective core-potential (ECP, LANL2DZ) for Pd, as implemented in the Spartan 10 (ver. 1.1.0, Wavefunction Inc., Irvine, CA 92612) computational package. Panels c,f,i,m show the molecular electrostatic potential mapped surfaces (MEPS), and panel **j** shows the spin density isosurface obtained from computation for the spin active Pd^{3+} specie, $S = 1/2$. All isosurfaces are drawn at 0.002 isoval. $<S^2>$ stands for expectation value.

Note: The colour maps given in panels **c**,**f**,**i**,**m** show the calculated MEPS surfaces (Molecular Electrostatic potential Mapped Surface) from optimized structures. Positive regions (coloured in blue) in the Pd mpg-CN model system illustrate areas where a deficiency of electron density exists. Such regions are localized

significantly around the Pd centre in all oxidation states tested (from 0 to 4+, labelled in the Figure as Pd(0), Pd(II), Pd(III), Pd(IV)), indicating that Pd is expected to interact with nucleophiles and/or negatively charged (donor) species (*e.g.*, OH^- from water). Conversely, the negative regions (coloured in red) correspond to areas containing an excess of electron density (localized all-over on the mpg-CN scaffold), and these regions interact with electrophiles or positively charged species. Upon increasing the oxidation state of Pd (from 0 to 4+), the distances Pd-N (N from the mpg-CN support) decrease as expected.

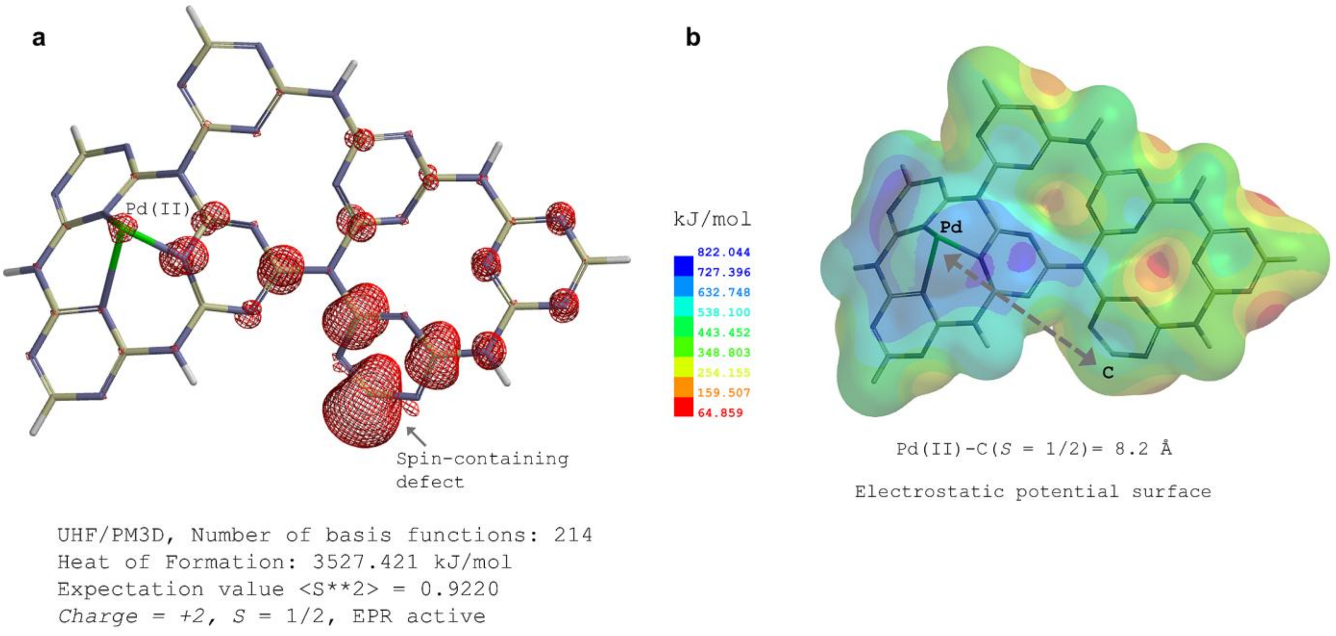

Figure S39. UHF/PM3(tm) model of an extended backbone array of carbon nitride units of the Pd-mpg-CN catalyst. The model shows (**a**) the presence of a spin containing defect in the carbon-nitride framework (indicated by an arrow) (EPR signal experimentally observed around *g* = 2.00) which expresses an effective spin polarization (the spin density isosurface is given in red in the panels) of the organic backbone perturbing the Pd^{2+} site (giving very weak signal at *g* = 2.085). This representation is fully consistent with the experimental EPR finding shown in Fig. 4A, main manuscript text. Panel (**b**) shows the MEPS surface.

To screen the conformational arrangements of the interacting systems (Pd-mpg-CN catalyst with TEA, TEA/OH^-, TEA/OH^-/Substrate) it was used combination of the Merk Molecular Force Field (MMFF94)/Monte Carlo methods. Restricted searches were applied using an energy window of E_{max} = 40 kJ mol^{-1} from a small pool of 3000 conformers. The best-scoring conformer was then used and refined using structural constraints from DFT data applied to the mpg-CN scaffold by geometry optimization via semiempirical quantum mechanical PM3(tm) method (for closed-shell, RHF; for open-shell, UHF). In the new Figure 40, as an example, it is shown the result from the conformational search of Pd-mpg-CN interacting with a TEA molecule and with bound hydroxyl group from water molecule. This species is EPR active (S = 1/2) and shows nitrogen hyperfine components from TEA, due to the coupling of the electron spin moment of Pd^{3+} with the N nucleus of TEA. The result is consistent with our observation of the EPR fingerprints of this system, obtained under ambient light and under *in-operando* light irradiation, given in the main text as Figure 4C water/1,4-dioxane/TEA mixture with *in situ* 325 nm irradiation, and as extended text in the Supporting Material (EPR analysis, Figure S33). Further details of such computed structures are given in the new Figure S40 and S41.

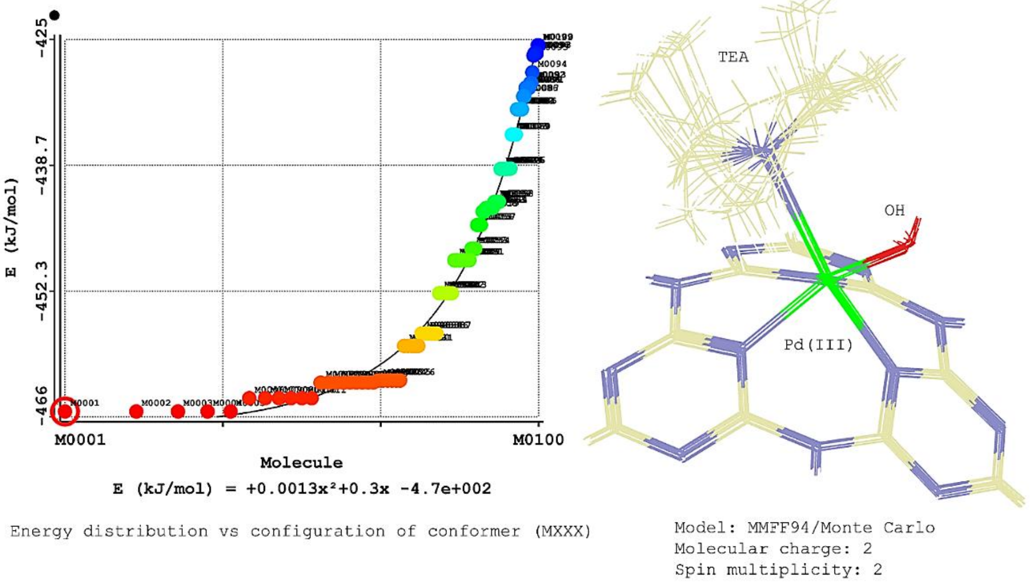

Figure S40. The results from the restricted (40 kJ/mol) conformational search of Pd-mpg-CN interacting with a TEA molecule and with bound hydroxyl group (OH^-) coming from water molecule.

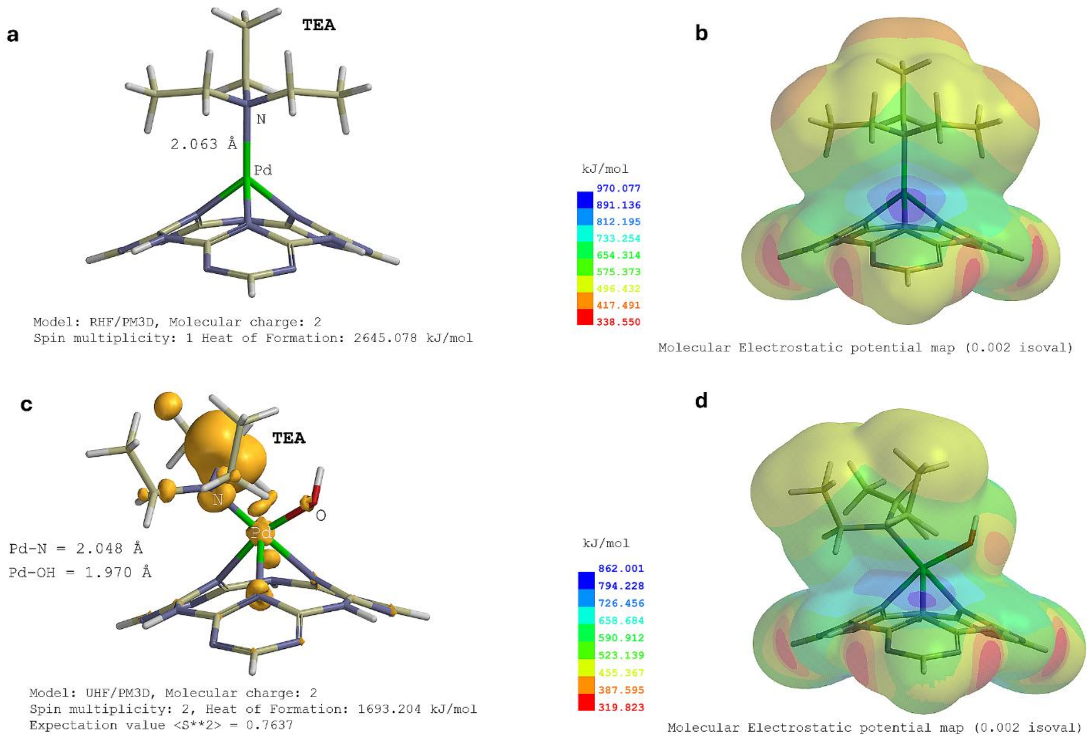

Figure S41. Semiempirical (PM3(tm)) models describing the interaction between Pd-mpg-CN with TEA molecule in the scenarios where a diamagnetic Pd centre is interacting with TEA (here taken as Pd^{2+}, panels **a**, optimized structure; panel **b**, the calculated MEPS surface) compared to the scenario in which Pd^{2+} is oxidized to Pd^{3+} in presence of bound OH^- from water (panel **c**, spin density isosurface; panel **d** the calculated MEPS surface). Note that panel **c** highlights the effective spin density shared between Pd with TEA molecule, in agreement with the experimental EPR observations of emergence of N hyperfine interactions and *g*-shift in resonance signals ($g_1 = 2.315$, $g_2 = 2.060$, $A_N = 17.8$ mT, shown in Figure S33).

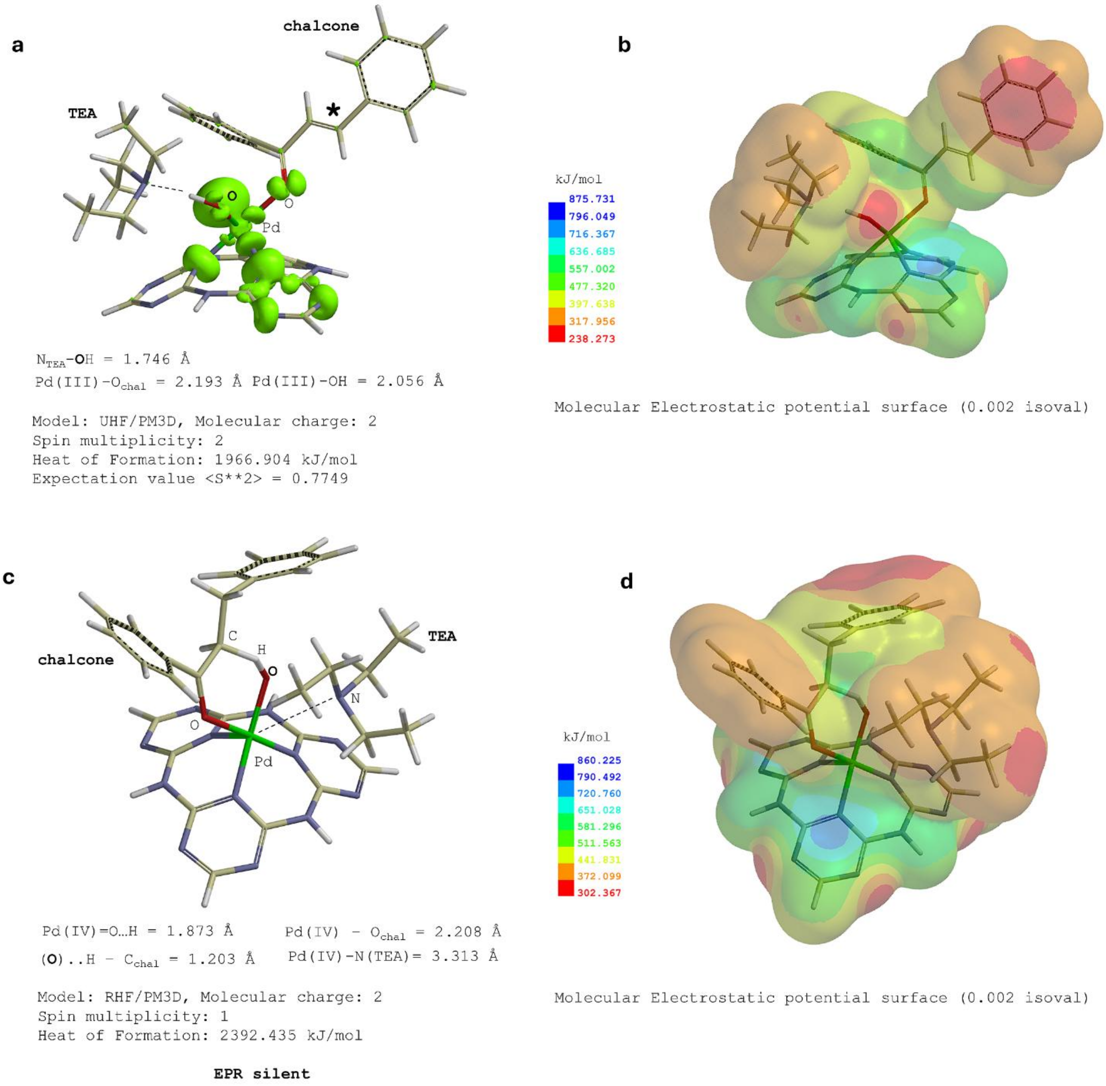

Figure S42. Structural models obtained by semiempirical calculations (PM3(tm)) of the single atom Pd-mpg-CN active site interacting with the chalcone substrate after reaction with one hole (panel **a**, generation of Pd^{3+}) or two holes (panel **c**, generation of Pd^{4+}) in presence of the electronic modulator TEA. Panels **b** and **d** show the corresponding MEPS surfaces.

Note: Upon interaction between Pd-mpg-CN with chalcone molecule (Pd^{3+} formation from oxidation of Pd^{2+} by photogenerated holes), the spin density is diverted towards the substrate, indicating stronger electronic communication with chalcone to TEA. As reported, TEA acts in such a scenario as an electronic modulator. Note that at this stage, not yet proton transfer has yet occurred to the carbon highlighted by the asterisk in panel a. Following proton transfer through spill-over of H^- generated at the Pd NPs sites to the previous carbon (*) and following further reaction with a second photogenerated hole, as given in the main reaction scheme in the manuscript text (Figure 5), Pd^{4+} is formed (panel c). At this stage, the interaction of

activated OH occurs and allows the occurrence of the second proton transfer (from OH) to chalcone. Both protons come from water molecules. Furthermore, interaction between TEA and Pd-*mpg*-CN further weakens, until Pd^{2+} is formed by ½ O_2 release, and then the catalytic cycle restarts.

References:

(1) J. Krzystek, J.-H. Park, M. W. Meisel, Mi. A. Hitchman, H. Stratemeier, L.-C. Brunel, J. Telser, *Inorg. Chem.* **2002**, 41, 4478-4487.
(2) J. Lati, J. Koresh, D. Meyerstein, *Chem. Phys. Lett.* **1975**, 33, 286-288.
(3) Pirovano, P.; Twamley, B.; McDonald, A. R. Modulation of Nickel Pyridinedicarboxamidate Complexes to Explore the Properties of High-valent Oxidants. *Chemistry – A European Journal* **2018**, 24, 5238-5245.
(4) Choudhury, S. B.; Lee, J.-W.; Davidson, G.; Yim, Y.-I.; Bose, K.; Sharma, M. L.; Kang, S.-O.; Cabelli, D. E.; Maroney, M. J. Examination of the Nickel Site Structure and Reaction Mechanism in Streptomyces seoulensis Superoxide Dismutase. *Biochemistry* **1999**, 38, 3744-3752.
(5) Geßner, C.; Trofanchuk, O.; Kawagoe, K.; Higuchi, Y.; Yasuoka, N.; Lubitz, W. Single crystal EPR study of the Ni center of NiFe hydrogenase. *Chemical Physics Letters* **1996**, 256, 518-524.
(6) Yu. Yu. Titov, L. B. Belykh and F. K. Schmidt. EPR Spectroscopy of catalytic systems based on nickel complexes of 1,4-diaza-1,3-butadiene (a-diimine) ligands in hydrogenation and polymerization reactions. *Low Temp. Phys.* **2015**, 41, 25-28. DOI:10.1063/1.4906313

Supplementary Tables

Table S1 Physicochemical properties of the catalysts.

Sample	$M^{[a]}$ (wt.%)	$S_{BET}^{[b]}$ (m^2 g^{-1})	$V_{pore}^{[c]}$ (cm^3 g^{-1})	$PD^{[d]}$ (nm)
mpg-CN	--	237	0.89	14.2
Pd-mpg-CN	2.9 (3.0)	176	0.84	15.3
Pt-mpg-CN	2.1 (3.0)	174	0.71	13.3
Au-mpg-CN	3.0 (3.0)	189	0.79	13.8
Ru-mpg-CN	2.9 (3.0)	190	0.79	13.9
Ni-mpg-CN	2.3 (3.0)	193	0.86	14.8

[a] ICP-OES (in parenthesis, theoretical loading). [b] BET method. [c] Pore volume at p/p_0 = 0.98. [d] Pore diameter.

Table S2 Structural parameters of the Pd-mpg-CN obtained by fitting the EXAFS spectrum.

Sample	Shell	$N^{[a]}$	$R^{[b]}$ (Å)	$\sigma^{2[c]}\times10^{-3}$ ($Å^2$)	$\Delta E_0^{[d]}$ (eV)	R factor[e]
Pd-mpg-CN	Pd-N/C	2.9±0.6	1.89±0.01	2.0	-6.4±0.8	0.002
	Pd-Pd	1.1±0.6	2.70±0.03			
Pd_1-mpg-CN	Pd-N/C	3.91±0.8	1.93±0.01	0.9	-0.8±2.4	0.014

[a] Coordination numbers. [b] Bond distance. [c] Debye-Waller factors. [d] Inner potential correction. [e] Goodness of fit.

Table S3 Photocatalytic transfer hydrogenation under various conditions.

Entry	Condition variations	Yield (%)
1	None[a]	95
2	Without light	n.d.[b]
3	Without water	n.d.
4	Without catalyst	n.d.
5	Without TEA	n.d.
6	Pd_{NP}-mpg-CN-1[c]	7.8%

[a] Reaction conditions: catalyst (10 mg), chalcone (0.1 mmol), ultrapure water (2 ml), 1,4-dioxane (3 ml), TEA (0.4 ml),

blue light 40 W (λ = 427 nm), reaction time (4 h), reaction temperature (313 K), reaction pressure (1 bar), N_2 atmosphere. [b] n.d.: not detected. [c] The Pd_{NP}-mpg-CN-1 was synthesized by the hydrogen reduction method at 773 K. Specifically, the mpg-CN (0.5 g) was added to deionized water (40 mL) under sonication for 1 h. Then, the metal precursor ($PdCl_2$, 3.0 wt.% metal relative to the carrier) was added and stirred overnight. The slurry was further stirred for 5 h at 333 K. The solids were collected by centrifuging, washed with deionized water and ethanol completely, and dried at 333 K overnight. Finally, it was treated at 773 K with a rate of 5 K/min for 5 h under a 10% H_2/Ar atmosphere.
